# Genome-wide identification of the *MADS-box* transcription factor family in pear (*Pyrus bretschneideri*) reveals evolution and functional divergence

**DOI:** 10.7717/peerj.3776

**Published:** 2017-09-11

**Authors:** Runze Wang, Meiling Ming, Jiaming Li, Dongqing Shi, Xin Qiao, Leiting Li, Shaoling Zhang, Jun Wu

**Affiliations:** Centre of Pear Engineering Technology Research, State Key Laboratory of Crop Genetics and Germplasm Enhancement, Nanjing Agricultural University, Nanjing, China

**Keywords:** Transcription factor, Functional divergence, Anthocyanin, Pear, MADS-box

## Abstract

*MADS-box* transcription factors play significant roles in plant developmental processes such as floral organ conformation, flowering time, and fruit development. Pear (*Pyrus*), as the third-most crucial temperate fruit crop, has been fully sequenced. However, there is limited information about the *MADS* family and its functional divergence in pear. In this study, a total of 95 *MADS-box* genes were identified in the pear genome, and classified into two types by phylogenetic analysis. Type I *MADS-box* genes were divided into three subfamilies and type II genes into 14 subfamilies. Synteny analysis suggested that whole-genome duplications have played key roles in the expansion of the *MADS* family, followed by rearrangement events. Purifying selection was the primary force driving *MADS-box* gene evolution in pear, and one gene pairs presented three codon sites under positive selection. Full-scale expression information for *PbrMADS* genes in vegetative and reproductive organs was provided and proved by transcriptional and reverse transcription PCR analysis. Furthermore, the *PbrMADS11(12)* gene, together with partners *PbMYB10* and *PbbHLH3* was confirmed to activate the promoters of the structural genes in anthocyanin pathway of red pear through dual luciferase assay. In addition, the *PbrMADS11* and *PbrMADS12* were deduced involving in the regulation of anthocyanin synthesis response to light and temperature changes. These results provide a solid foundation for future functional analysis of *PbrMADS* genes in different biological processes, especially of pigmentation in pear.

## Introduction

Transcription factors are usually defined as proteins that activate and/or repress gene transcription by binding to sequence-specific DNA, and play critical roles in controlling biological processes (Riechmann et al., 2000). A typical plant transcription factor generally contains a DNA-binding region, a transcription-regulation domain, an oligomerization site, and a nuclear localization signal (Liu, White & MacRae, 1999). In addition, transcription factors usually belong to large multigene families, and show high complexity of transcriptional regulation (Riechmann et al., 2000). *MADS-box* transcription factors are widely distributed in eukaryotes, and have been isolated from plants, animals and fungi (Messenguy & Dubois, 2003). In plants, *MADS-box* genes can be divided into type I and type II by evolutionary relationships (Alvarez-Buylla et al., 2000). In general, type I proteins contain conserved MADS (M) domains (Parenicova et al., 2003), and are divided into three subfamilies: Mα, Mβ, and Mγ. Type II proteins differ from type I in that they include four domains from N to C terminus: the MADS (M), the Intervening (I), the Keratin (K), and the C-terminal (C) domains (Kaufmann, Melzer & Theißen, 2005). The M domain, containing about 60 amino acids, is the most conserved domain for DNA binding (Shore & Sharrocks, 1995; Melzer, Wang & Theissen, 2010). The mid-level conserved K domain has a coiled-coil structure of approximately 70 amino acids and is involved in protein-protein interaction (Riechmann, Krizek & Meyerowitz, 1996). The I domain takes part in the formation of a specific DNA-binding dimer (Davies et al., 1996; Riechmann & Meyerowitz, 1997). The most variable domain, C, mainly contributes to transcription activation (Kramer, Dorit & Irish, 1998). Type II proteins can be further classified into two types: MIKC^c^ and MIKC^∗^, according to the differences of gene structure. Compared with MIKC^c^ proteins, MIKC^∗^ proteins tend to have a longer I domain and a less conserved K domain (Henschel et al., 2002). Based on phylogenetic relationships, MIKC^c^ *MADS-box* genes can be further subdivided into 12 subfamilies in *Arabidopsis* (Becker & Theißen, 2003). Comparatively, type I genes experience a faster birth and death rate compared with type II genes (Parenicova et al., 2003; Nam et al., 2004).

*MADS-box* transcription factors play significant roles in plant development processes. One of their most important roles is in floral organ identity (Alvarez-Buylla et al., 2000). The ‘ABCDE’ genetic model explains how A, B, C, D, and E function genes determine floral organs. A and E are required for sepals, A, B, and E for petals, B, C, and E for stamens, C and E for carpels, and D and E for ovules (Coen & Meyerowitz, 1991; Weigel & Meyerowitz, 1994; Gutierrez-Cortines & Davies, 2000; Honma & Goto, 2001; Zahn, Feng & Ma, 2006). In *Arabidopsis*, A, B, C, D, and E function clades correspond to genes from *AP1 (APETALA1), AP3/PI (APETALA3/PISTILATA), AG (AGAMOUS), STK/AGL11 (SEEDSTICK/AGAMOUS-LIKE11)*, and *SEP (SEPALLATA)* subfamilies. Besides their functions in floral organ identity, *MADS-box* genes are also involved in the control of flowering time (*FLOWERING LOCUS C*: *FLC*, *SHORT VEGETATIVE PHASE*: *SVP*, *SUPPRESSOR OF OVEREXPRESSION OF CONSTANS1*: *SOC1* and *FRUITFULL*: *FUL* genes), fruit development (*SHATTERPROOF*: *SHP*, *AG*, *and AP1/FUL* genes), endodormancy (*dormancy-associated MADS-box*: *DAM* genes), root development (*AGL12* and *AGL17* genes) (Rodriguez et al., 1994; Michaels & Amasino, 1999; Ferrandiz, Liljegren & Yanofsky, 2000; Hartmann et al., 2000; Liljegren et al., 2000; Samach et al., 2000; Tapia-López et al., 2008), and pigment accumulation (*TRANSPARENT TESTA 16:TT16*) (Causier, Kieffer & Davies, 2002; Nesi et al., 2002; Wu et al., 2013b).

For pear *MADS-box* genes, more research has been done in flower bud dormancy. Two dormancy-associated *MADS-box* (DAM) genes have been isolated from *P. pyrifolia*, and their expression patterns during the seasonal endodormancy transition phases have been reported (Ubi et al., 2010). Two independent transcriptomics-based analyses of pear buds have provided valuable resources for the *MADS-box* gene identification associated with dormancy regulation (Liu et al., 2012; Bai et al., 2013). Moreover, 30 MIKC^c^-type *MADS-box* genes, including *PpMADS13*, were identified and characterized during flower bud dormancy in pear (Niu et al., 2016; Saito et al., 2013; Saito et al., 2015). The functions of *MADS-box* genes in development of flower and fruit have also been reported. For example, an *AP1-like* (*APETALA1-like*) gene was identified in reproductive organ development in Japanese pear (*P. pyrifolia*) (Liu et al., 2013b), while ten *MADS-box* genes were cloned in *P. pyrifolia*, with their expression during fruit development and ripening analyzed (Ubi et al., 2013).

Because of the critical regulatory functions of *MADS-box* genes in plant responses to different developmental processes, the *MADS-box* gene family has been extensively studied in the model plant *Arabidopsis thaliana*, as well as in non-model plants such as rice (*Oryza sativa*), maize (*Zea mays*), poplar (*Populus trichocarpa*), and apple (*Malus* ×* domestica*) (Parenicova et al., 2003; Arora et al., 2007; Zhao et al., 2011; Leseberg et al., 2006; Tian et al., 2015). However, to date, no genome-wide characterization of the *MADS* family has been conducted in pear. Pear is the third-most crucial temperate fruit crop (Wu et al., 2013a), and belongs to the *Pomaceae* subfamily in Rosaceae. The genome of ‘Dangshansuli’ (*P. bretschneideri*) has been sequenced recently (Wu et al., 2013a), which allows for analysis of the *MADS-box* transcription factor family. In this paper, we identified *MADS-box* genes across the pear genome. Phylogenetic, gene structural, conserved motif, synteny and positive selection analyses were also carried out. Expression patterns of *MADS-box* genes in eight vegetative and reproductive organs were further surveyed. *MADS-box* genes that might be related to anthocyanin accumulation were verified using qRT-PCR and dual luciferase assay. These data provide a solid foundation for future functional analysis of *PbrMADS* genes in different biological processes, especially for pigmentation related *MADS-box* genes.

## Materials and Methods

### Identification of *MADS-box* genes in pear

The genome sequence files of pear were downloaded from the Pear Genome Project (http://peargenome.njau.edu.cn) (Wu et al., 2013a). The full-length *MADS-box* protein sequences of *Arabidopsis* and rice were downloaded from The Arabidopsis Information Resource (TAIR) (http://www.arabidopsis.org) and the Rice Genome Annotation Project (RGAP) (http://rice.plantbiology.msu.edu/) as previously described, respectively (Parenicova et al., 2003; Arora et al., 2007). To identify members of the *MADS-box* transcription factor family in pear, two strategies were used: Hidden Markov Model search (HMM search) with the MADS domain HMM profile (PF00319) and BLASTP searches using *MADS-box* protein sequences from *Arabidopsis* and rice as queries. Firstly, the keyword ‘MADS’ was used in the Pfam database (Finn et al., 2010) to find the MADS domain seed alignment file (PF00319). A HMM was built using the seed alignment file by HMMER software package (version 3.0) (Eddy, 2011) and HMM searches were performed against the local protein database of pear using HMMER with an *E*-value threshold of 1e^−1^. Secondly, *MADS* protein sequences from *Arabidopsis* and rice were used as queries to perform BLASTP searches against pear protein database with an *E*-value cutoff of 1e^−1^. We initially checked the chromosome localizations and removed redundant sequences with the same physical location to obtain candidate proteins. Then, these proteins were submitted to NCBI CDD (Conserved Domain Database, http://www.ncbi.nlm.nih.gov/Structure/cdd/wrpsb.cgi) (Marchler-Bauer et al., 2015) to confirm the presence and completeness of the MADS domain (*E*-value threshold 1e^−2^). Protein sequences with MADS domain were further inspected using SMART (Simple Modular Architecture Research Tool, http://smart.embl-heidelberg.de/) (Letunic, Doerks & Bork, 2012) (*E*-value threshold 1e^−2^, with manual inspection of sequences close to the threshold). Protein sequences lacking the MADS domain or having *E*-value beyond 1e^−2^ for *MADS* domain in SMART analyses were removed in the following analyses.

### Phylogenetic analysis of the *MADS-box* transcription factor family

We used two statistical methods to construct the phylogenetic trees: neighbor-joining (NJ) method and maximum-likelihood (ML) method. For the NJ method, sequence alignments were performed using MUSCLE program in MEGA6 (Tamura et al., 2013) with default parameters and refined manually. Then, an NJ (neighbor-joining) phylogenetic tree was generated using MEGA6 with a P-distance model, the pairwise deletion of gaps, and bootstrap analysis with 1,000 replicates. For the ML method, multiple sequence alignment was executed using MAFFT software (version 7.03) (Katoh & Standley, 2013) and refined manually, and substitution model matching was performed using Model Generator tool (version 0.85) (Keane et al., 2006). The ML tree was constructed using the RAxML toolkit (version 8.0) (Stamatakis, 2014) with a matched JTT model and 100 bootstrap replications. The *MADS-box* family is a big gene family, with two types of genes (Type I and Type II) that are quite different. It is difficult and inaccurate to classify them into specific subfamilies in one tree because of low bootstrap values caused by sequence differences. Therefore, we pre-classified them into two types, and *Arabidopsis MADS* genes were used to assist classification. Pear *MADS-box* genes that clustered together with *Arabidopsis* type I and type II genes were classified as type I and type II genes, respectively. Furthermore, phylogenetic trees of type I and type II genes were constructed independently for detailed classification of subfamilies, together with *Arabidopsis* and rice as the reference.

### Gene structure and conserved motif analysis of the *MADS-box* Genes

Gene structures of *MADS-box* genes were extracted from released GFF (General Feature Format) file (http://peargenome.njau.edu.cn) and drawn using GSDS (Gene Structure Display Server, http://gsds.cbi.pku.edu.cn) (Hu et al., 2015). Conserved motifs were identified using MEME (version v.4.9.1) (Multiple EM for Motif Elicitation, http://meme-suite.org/tools/meme) (Bailey & Elkan, 1994) with the following parameters: any number of repetitions; 20 different motifs, motif width of 6–200 amino acids.

### Chromosomal locations and synteny analysis

Genome annotation files were downloaded from the pear genome database to obtain chromosomal location information of the *MADS-box* genes. Circos software (Krzywinski et al., 2009) was then used to draw the location picture. A method similar to that developed for the PGDD (Plant Genome Duplication Database, http://chibba.agtec.uga.edu/duplication/) (Lee et al., 2013) was used to conduct synteny analysis of the pear genome. First, BLASTP was used to search potential homologous sequences (*E*-value < 1e^−5^, top 5 matches) in the pear genome. Then, MCScanX (Wang et al., 2012) was used to identify syntenic regions by inputting homologous sequences. Finally, syntenic regions valuation was performed using Colinear Scan procedure with an *E*-value of <1e^−10^. MCScanX was further used to detect WGD (Whole-genome duplication) or segmental, tandem and dispersed duplicates retained in the *MADS-box* transcription factor family (Johansen et al., 2002).

### Ka and Ks calculations and tests of positive selection

To reveal the date of segmental duplication events, homologous gene pairs in the 100 kb flanking each side of the *PbrMADS* genes were chosen to estimate the mean Ks. MEGA6 was used to make the pairwise alignments of the homologous nucleotide coding sequences, with the corresponding protein sequences as the alignment guides. Nonsynonymous (Ka) and synonymous (Ks) substitution rates were calculated using the program KaKs_Calculator 2.0 with the NG method (Wang et al., 2010). The mean Ks values were then used to calculate the approximate date of the duplication event. Moreover, the branch-site model method was used to detect the codon sites of positive selection for paralogous gene pairs in the PAML software package (Yang & Dos Reis, 2011). Phylogenetic trees of pear and apple *MADS* genes (Kumar et al., 2016) for the branch-site model were constructed using ML and NJ methods. Genes with different topologies between the methods and located on low bootstrap branches (<50) were removed. Then, a new phylogenetic tree was reconstructed by ML method and the tree topology was further confirmed using the NJ method. Node for each paralogous pair was designated as the foreground branch and the others as background branches, respectively. The alternative model A (positive selection, model =2, NS sites = 2, and fix_omega = 0) was compared with the null model A1 (neutral selection, model = 2, NS sites = 2, and fix omega = 1) to find codon sites under probable positive selection in our study. Each test was run applying four different starting values for omega estimates for site classes under positive selection (0.5, 1, 1.5, and 2) and the results from the analyses with highest likelihood scores were used (Yang & Dos Reis, 2011; Vigeland et al., 2013). LRT (likelihood ratio test) was used to compare the two models to see the omega ratio difference among lineages. Correction for multiple testing was performed using false discovery rate with the p.adjust function in R, over all *P*-values, treated as one series of repetitions (Proux et al., 2009). Positive selection is indicated if the alternative model is significantly better than the null model at the 5% level (FDR cut-off value). Finally, the BEB (Bayes Empirical Bayes) method was used to identify codon sites under probable positive selection and genes with positive selection at 5% level (Yang, Wong & Nielsen, 2005).

### Plant materials, anthocyanin measurement, RNA extraction and first-strand cDNA synthesis

Young root, young stem, mature leaf, young leaf, flower, young fruit, style, and pollen were sampled from the pear cultivar *P. bretschneideri* grown in the Jiangpu Orchard of Nanjing Agricultural University. Unexpanded young leaves were collected a few days after leaf bud breaking in pear trees in the orchard, while the mature leaves were harvested after 3 weeks after bud breaking. Flowers were collected few days before anthesis, young fruits were collected 15 days after full blooming (DAFB). As pear trees in the orchard used rootstock, young roots and stems were collected from germinated seeds. Young roots and stems were harvested at 50 days after seed germination and transferred to pots containing soil and vermiculite. Fruits of the red-colored ‘Starkrimson’ (previous named ‘Early red Doyenne du Comice’, *P. communis*) and its green variant strain (previous named ‘Green Doyenne du Comice’) were sampled from the pear orchard of the Changli Institute of Pomology, Hebei Academy of Agriculture and Forestry Sciences of China. Green variant strain originated from Co^60^-γ mutagenesis of ‘Starkrimson’ and had been stabilized for five years. Pear fruits at different developmental stages were collected from fruit set to fruit maturation in 2013, specifically, at fruit early enlargement stage (40 DAFB), fruit rapid enlargement stage (55 DAFB), a month after fruit enlargement stage (70 DAFB), and pre-mature stage (85 DAFB). The fruits of ‘Hongzaosu’ (*P. bretschneideri*) were collected from experimental orchard of the College of Horticulture at Nanjing Agricultural University. Fruits of uniform size and growing stages were selected for bagging treatment, and non-bagged fruits were used for the control. All fruits were harvested about 15 days before commercial maturity. The bagging fruits were debagged and randomly divided into two groups and placed at different temperature conditions: high temperature (HT, 30 °C) and low temperature (LT, 17 °C), both groups under same light condition of UV-B/visible light irradiation (Ubi et al., 2006). Fruit samples were collected at 4 d, 8 d and 12 d after treatment. For each sample, the skin of fruits was peeled off and immediately placed in liquid nitrogen and stored at −70 °C before isolation of total RNA.

The fruit skin (1g) was used to extract anthocyanin in 5 mL 1% HCl-methanol solution at 4 °C for 24 h. After centrifugation at 12,000 g for 20 min, a UV-vis spectrophotometer (MAPADA UV-1800; Shanghai Mapada Instruments, Shanghai, China) was used to observe the upper aqueous phase at 530, 620, and 650 nm. The relative anthocyanin content was calculated using the following formula: OD = (A530 − A620) − 0.1(A650 − A620) (Lee & Wicker, 1991). One unit of anthocyanin content was defined as 0.1 OD change (unit  ×10^3^ g^−1^ FW). For each sample three replications were analyzed.

Total RNA was extracted from harvested materials using the Plant Total RNA Isolation Kit (Chengdu Foregene Biotech Technology Co., Ltd, Chengdu, China). 1% agarose gel electrophoresis was used to assess RNA integrity, and the concentration of extracted RNA was determined by NanoDrop (Thermo Fisher Scientific, Waltham, MA, USA). Finally, the first-strand cDNA was synthesized from total RNA with m-MLV (TransGen, Beijing, China) in accordance with the manufacturer’s protocol.

### RT-PCR and qRT-PCR

Reverse transcription PCR (RT-PCR) was used to quantify the transcript expression of *PbrMADS* genes in vegetative and reproductive organs. Reactions were executed using Taq DNA Polymerase (Sangon Biotech, Shanghai, China) and 300 ng cDNA from each sample. The thermal cycling conditions were 94 °C for 3 min, 35 cycles of 94 °C for 30s, 56 °C for 30 s, 72 °C for 30 s, and final extension at 72 °C for 10 min. Amplification products were detected by 2% agarose gel. Specific primers were designed for *PbrMADS* genes; for those CDS (coding sequence) regions with high similarity, the UTR (untranslated region) sequences were also used for primer design; however, for six pairs of gene (*PbrMADS1* and *PbrMADS2*, *PbrMADS15* and *PbrMADS16*, *PbrMADS20* and *PbrMADS21*, *PbrMADS24* and *PbrMADS25*, *PbrMADS92* and *PbrMADS93*, and *PbrMADS94* and *PbrMADS95*), we could not find appropriate primers because of high similarity both in CDS and UTR. Therefore, the transcript level of each highly similar gene pair was detected by the same primer pair (Table S1). *Pyrus* Tubulin (Tubulin, accession number AB239681) was used as a standard gene for different gene expressions.

Real-time quantitative RT-PCR (qRT-PCR) was performed using LightCycler 480 (Roche, USA). For each reaction mixture, the volume was 20 µl, containing 10 µl LightCycler 480 SYBR GREEN I Master (Roche, Indianapolis, IN, USA), 0.5 µl of diluted cDNA, 5 µl of each gene-specific primer, and 4.5 µl nuclease-free water. The PCR reaction conditions were set as follows: pre-incubation at 95 °C for 10 min and then 55 cycles of 94 °C for 3 s, 60 °C for 10s, 72 °C for 30 s, and a final extension at 72 °C for 3 min. Fluorescence was measured at the end of each annealing step. A melting curve analysis was performed from 60 °C to 95 °C in order to verify the specificity of each primer combination. *Pyrus* Tubulin (Tubulin, accession number AB239681) was used as an internal control to normalize the quantitative expression for all selected genes. Relative expression levels were quantified with the comparative Delta-delta Ct (threshold cycle) method (Livak & Schmittgen, 2001). qPCR data has three replicates.

### Expression analysis using EST data

The EST (expressed sequence tag) data was obtained from a mixed system of 12 different tissues including stems, leaves, fruits, flowers, and seeds at different stages of development from pear cultivar ‘Dangshansuli’ (*P. bretschneideri*) (Wu et al., 2013a). We retrieved the ESTs from the pear genome project (http://peargenome.njau.edu.cn). A local BLASTN was performed against pear EST libraries to get the hits for each *MADS-box* genes. Parameters were set as follow: maximum target sequences = 200 bp, and *E*-value <10^−10^.

### Excavation of *MADS-box* genes related to anthocyanin accumulation and regulation

*MADS-box* genes reported to be involved in anthocyanin accumulation and regulation were collected and their protein sequences were retrieved from NCBI, according to corresponding accession numbers. Then, these protein sequences and identified pear *MADS* genes were put together to construct a phylogenetic tree using MEGA6. Genes clustered in the same clade with anthocyanin related genes were considered to be candidates participating in anthocyanin accumulation and regulation in pear. Furthermore, qRT-PCR was used to verify the validity of candidate genes. Seven structural genes in the anthocyanin biosynthesis pathway cloned in our previous study (Yang et al., 2013), were used to analyze their cis-elements. First, the sequences of these genes were obtained from NCBI according to their accession numbers (KC460392, KC460393, KC460394, KC460395, KC460396, KC460397, and KC460398). Then, BLASTN searches were executed against the ‘Bartlett’ (*P. communis*) genome database (Chagné et al., 2014) for corresponding gene names and locations. Finally, 3 kb upstream promoter sequences of these genes were retrieved from genome database and subjected to PLACE (Plant cis-acting regulatory DNA elements database, http://www.dna.affrc.go.jp/PLACE) to identify the presence of MADS-binding cis-motifs (CArG-box) (Higo et al., 1999). The MADS-binding sites for promoter regions of R2R3-MYB genes in pear were also detected by PLACE.

### Dual luciferase assay of transiently transformed *Arabidopsis* protoplast

Dual luciferase assay was conducted using *Arabidopsis* mesophyll protoplasts as previously described (Yoo, Cho & Sheen, 2007). *Arabidopsis* grown on soil with a short photoperiod (8 h light/16 h dark at 22 °C), 4-week-old leaves were used to isolate protoplasts. Promoter sequences (2 kb upstream of the initiation codon) of *PbDFR1*, *PbUFGT1* and *PbANS1* were amplified from ‘Starkrimson’ and inserted into a pGreenII 0800–LUC vector. The full-length coding sequences of *PbrMADS11*, *PbrMADS12*, *PbMYB10* and *PbbHLH3* were inserted into pGreenII 62-SK vectors under the 35S promoter. Empty pGreenII 0800–LUC vector served as a negative control. Plasmid was extracted using the Plasmid Maxprep Kit (Vigorous Biotechnology, Taichung City, Taiwan). The Dual Luciferase Reporter Assay System (Promega, Madison, WI, USA) was used to determine the relative expression of Luc:Ren. Luc/Ren activity was measured in a microplate reader (Tecan Infinite M200).

## Results and Discussion

### Identification of *MADS-box* genes in pear

To identify the *MADS* gene family, we searched for genes that encode proteins with the *MADS* DNA-binding domain across the whole genome sequence of pear. The seed file of *MADS* domain (PF00319) from Pfam (http://pfam.janelia.org/) was used to obtain the HMM (Hidden Markov Model) sequence file, then HMM searches were performed in HMMER3.0 software against the pear protein database (http://peargenome.njau.edu.cn/). We also used the *Arabidopsis* and rice *MADS* protein sequences as queries to perform BLASTP searches against the pear genome databases. A total of 121 candidate *MADS* genes were identified. We removed 24 genes due to non-existence or incompleteness of a *MADS* domain. A further two candidates were removed for containing many additional domains, with no *MADS-box* homologs of other organisms. Finally, 95 nonredundant and complete *MADS-box* genes in the pear genome were collected for further analysis (Table 1). We named them *PbrMADS1* through *PbrMADS95* based on guidelines for gene naming in Rosaceae (Jung et al., 2015).

**Table 1:** The *MADS-box* transcription factors identified in pears.

Gene name	Gene ID	Chr locus	Genomic position	Protein length (aa)	K domain (Y/N)	EST hits (Y/N)	Type
PbrMADS1	Pbr035643.1	13	5802860–5807937	240	Y	Y	MIKC^c^
PbrMADS2	Pbr015153.1	16	5932222–5938012	251	Y	Y	MIKC^c^
PbrMADS3	Pbr016601.1	17	17647798–17647986	63	N	Y	MIKC^c^
PbrMADS4	Pbr022183.1	9	18587853–18588041	63	N	Y	MIKC^c^
PbrMADS5	Pbr023545.1	6	21412278–21417880	240	Y	Y	MIKC^c^
PbrMADS6	Pbr029989.1	13	4385598–4392040	307	Y	Y	MIKC^c^
PbrMADS7	Pbr020185.1	6	4424778–4429823	249	Y	Y	MIKC^c^
PbrMADS8	Pbr020186.1	6	4405924–4406359	74	N	Y	MIKC^c^
PbrMADS9	Pbr018801.2	2	603223–614105	668	Y	Y	MIKC^c^
PbrMADS10	Pbr008076.1	sffold1479.0	33652–33879	76	N	Y	MIKC^c^
PbrMADS11	Pbr016599.2	17	17667140–17673579	222	Y	Y	MIKC^c^
PbrMADS12	Pbr007180.1	14	15043269–15048861	256	Y	Y	MIKC^c^
PbrMADS13	Pbr029990.1	13	4377373–4381393	240	Y	Y	MIKC^c^
PbrMADS14	Pbr036879.1	14	13999288–14002989	224	Y	Y	MIKC^c^
PbrMADS15	Pbr037444.1	6	18929–21076	170	Y	Y	MIKC^c^
PbrMADS16	Pbr017715.1	6	437472–439619	200	Y	Y	MIKC^c^
PbrMADS17	Pbr039900.1	sffold867.0	119095–119372	63	N	Y	MIKC^c^
PbrMADS18	Pbr001551.1	6	14880923–14887496	138	N	Y	MIKC^c^
PbrMADS19	Pbr039897.1	sffold867.0	76730–76921	64	N	Y	MIKC^c^
PbrMADS20	Pbr001458.1	sffold1032.0	108284–109564	117	N	Y	MIKC^c^
PbrMADS21	Pbr001460.1	sffold1032.0	121179–122459	117	N	Y	MIKC^c^
PbrMADS22	Pbr013902.1	7	12939336–12970807	239	Y	Y	MIKC^c^
PbrMADS23	Pbr032788.1	1	8123761–8132448	236	Y	Y	MIKC^c^
PbrMADS24	Pbr032787.2	1	8143132–8159638	254	Y	Y	MIKC^c^
PbrMADS25	Pbr001457.1	sffold1032.0	93002–97487	239	Y	Y	MIKC^c^
PbrMADS26	Pbr022146.1	15	19423131–19425153	241	Y	Y	MIKC^c^
PbrMADS27	Pbr040541.1	2	15725115–15727317	235	Y	Y	MIKC^c^
PbrMADS28	Pbr035294.1	8	7359228–7362459	216	Y	Y	MIKC^c^
PbrMADS29	Pbr029686.2	9	13855887–13862738	243	Y	Y	MIKC^c^
PbrMADS30	Pbr039503.1	10	7101939–7110441	244	Y	Y	MIKC^c^
PbrMADS31	Pbr000556.1	5	24521775–24530180	246	Y	Y	MIKC^c^
PbrMADS32	Pbr004239.1	8	5533059–5533244	62	N	Y	MIKC^c^
PbrMADS33	Pbr000828.1	15	40714549–40721545	225	Y	Y	MIKC^c^
PbrMADS34	Pbr002033.1	14	7643260–7648572	267	Y	Y	MIKC^c^
PbrMADS35	Pbr025860.1	3	2235923–2242424	323	Y	Y	MIKC^c^
PbrMADS36	Pbr009670.1	7	1511095–1515332	258	Y	Y	MIKC^c^
PbrMADS37	Pbr022918.2	2	7101509–7106026	258	Y	Y	MIKC^c^
PbrMADS38	Pbr040108.1	sffold872.0	38975–39447	90	N	N	MIKC^c^
PbrMADS39	Pbr007029.1	5	989690–989920	77	N	N	MIKC^c^
PbrMADS40	Pbr036758.1	5	5383182–5383400	73	N	N	MIKC^c^
PbrMADS41	Pbr007915.1	7	8282013–8282231	73	N	N	MIKC^c^
PbrMADS42	Pbr007481.1	15	38293055–38293246	64	N	N	MIKC^c^
PbrMADS43	Pbr019340.1	8	967796–978558	234	Y	Y	MIKC^c^
PbrMADS44	Pbr038022.1	15	38658322–38668704	123	N	Y	MIKC^c^
PbrMADS45	Pbr029333.1	sffold491.0	141754–142664	81	N	Y	MIKC^c^
PbrMADS46	Pbr019339.1	8	938405–938686	94	N	Y	MIKC^c^
PbrMADS47	Pbr003650.1	13	10532036–10535566	225	Y	Y	MIKC^c^
PbrMADS48	Pbr039693.1	15	26890960–26894582	225	Y	Y	MIKC^c^
PbrMADS49	Pbr021448.1	10	2035089–2037780	260	Y	Y	MIKC^c^
PbrMADS50	Pbr004234.1	8	5488881–5495600	203	Y	N	MIKC^c^
PbrMADS51	Pbr000804.1	15	40943666–40950346	189	Y	N	MIKC^c^
PbrMADS52	Pbr042160.2	15	33038074–33044155	348	N	Y	MIKC*
PbrMADS53	Pbr022012.1	8	12737277–12740508	303	N	Y	MIKC*
PbrMADS54	Pbr007292.1	14	15821857–15830381	809	N	Y	MIKC*
PbrMADS55	Pbr011423.3	6	1824609–1827345	375	N	Y	MIKC*
PbrMADS56	Pbr039074.1	13	2703781–2707742	438	N	Y	MIKC*
PbrMADS57	Pbr025656.1	10	16499696–16500379	228	N	N	Mα
PbrMADS58	Pbr034610.1	5	7051644–7052366	241	N	N	Mα
PbrMADS59	Pbr039562.1	10	6671771–6672298	176	N	N	Mα
PbrMADS60	Pbr025657.1	10	16496114–16496827	238	N	N	Mα
PbrMADS61	Pbr039561.1	10	6674815–6675441	209	N	N	Mα
PbrMADS62	Pbr018829.1	2	237735–238322	196	N	N	Mα
PbrMADS63	Pbr025970.1	sffold417.0	29167–29871	235	N	N	Mα
PbrMADS64	Pbr025981.1	sffold417.0	377815–378519	235	N	N	Mα
PbrMADS65	Pbr029054.1	9	8250784–8251095	104	N	Y	Mα
PbrMADS66	Pbr027548.1	9	10296082–10296783	234	N	N	Mα
PbrMADS67	Pbr033409.1	17	13872712–13873416	235	N	N	Mα
PbrMADS68	Pbr033418.1	17	13722980–13723684	235	N	N	Mα
PbrMADS69	Pbr031473.1	3	9785284–9786327	348	N	Y	Mα
PbrMADS70	Pbr001328.1	12	18235312–18235986	225	N	N	Mα
PbrMADS71	Pbr003216.1	sffold1135.0	13074–14075	334	N	N	Mβ
PbrMADS72	Pbr026551.1	8	4050787–4051728	314	N	N	Mβ
PbrMADS73	Pbr022939.1	2	6904265–6904744	160	N	N	Mβ
PbrMADS74	Pbr037101.1	17	4143639–4144730	364	N	N	Mβ
PbrMADS75	Pbr032195.1	8	6410894–6411958	355	N	N	Mβ
PbrMADS76	Pbr004263.1	8	5777631–5778242	204	N	Y	Mβ
PbrMADS77	Pbr031262.1	15	39184380–39185214	106	N	N	Mβ
PbrMADS78	Pbr009640.1	sffold160.2	126297–127616	440	N	N	Mγ
PbrMADS79	Pbr030435.1	10	15484934–15485575	214	N	N	Mγ
PbrMADS80	Pbr010321.1	14	1878629–1879402	258	N	N	Mγ
PbrMADS81	Pbr004617.1	sffold1211.0	68578–68904	109	N	N	Mγ
PbrMADS82	Pbr036986.1	sffold740.0	65837–66163	109	N	N	Mγ
PbrMADS83	Pbr036992.1	sffold740.0	145135–145461	109	N	N	Mγ
PbrMADS84	Pbr006795.1	6	18621477–18621950	109	N	N	Mγ
PbrMADS85	Pbr006798.1	6	18614500–18615171	224	N	N	Mγ
PbrMADS86	Pbr006794.1	6	18630739–18631368	210	N	N	Mγ
PbrMADS87	Pbr019318.1	8	641097–641864	256	N	N	Mγ
PbrMADS88	Pbr008912.1	sffold1558.0	8984–9301	106	N	N	Mγ
PbrMADS89	Pbr005990.1	16	10121071–10121748	226	N	N	Mγ
PbrMADS90	Pbr026074.1	12	3872966–3873643	226	N	N	Mγ
PbrMADS91	Pbr006693.1	4	2422773–2423276	168	N	N	Mγ
PbrMADS92	Pbr005991.1	16	10125235–10125795	187	N	Y	Mγ
PbrMADS93	Pbr026073.1	12	3868978–3869538	187	N	Y	Mγ
PbrMADS94	Pbr026075.1	12	3878308–3878949	214	N	Y	Mγ
PbrMADS95	Pbr005989.1	16	10115643–10116284	214	N	Y	Mγ

### Classification and phylogenetic analysis of *MADS-box* family genes in pear

**Figure 1:**
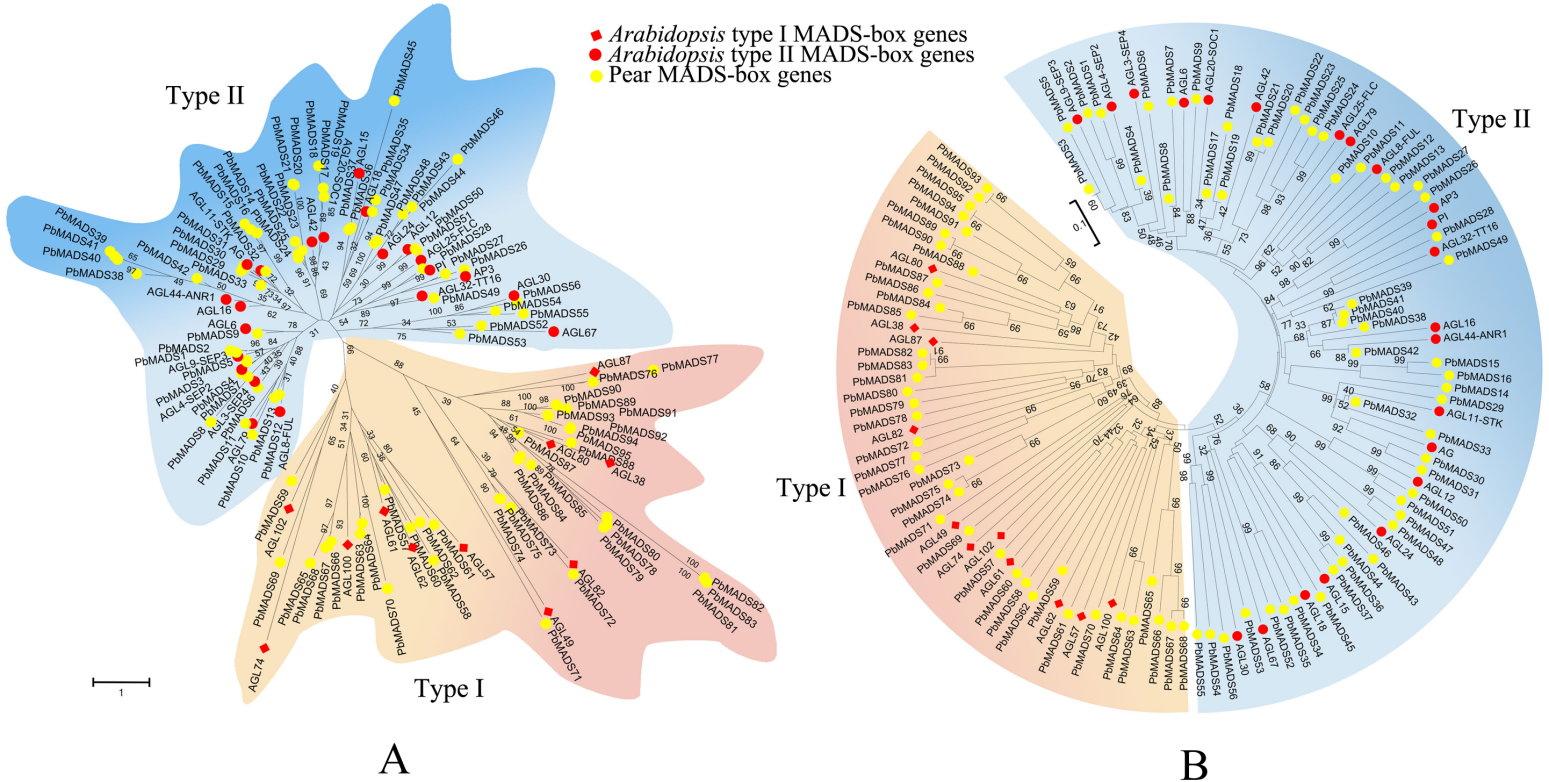
Phylogenetic trees of pear and *Arabidopsis MADS-box* proteins. (A) A phylogenetic tree generated by maximum-likelihood method. (B) A phylogenetic tree made by neighbor-joining method. A total of 33 representative *MADS-box* genes from different subfamilies of *Arabidopsis* were used. These trees are classified into two clades, designated as type I and type II.

To pre-classify pear *MADS-box* proteins into different types, two strategies were used: Neighbor-joining (NJ) method using MEGA6 and Maximum-likelihood (ML) method using RAxML. We first classified *MADS-box* genes of pear into two types as in *Arabidopsis* (Parenicova et al., 2003). According to the NJ phylogenetic tree (Fig. 1), 39 genes that clustered together with *Arabidopsis* type I genes were labeled as type I (containing the Mα, M β, and Mγ clades) and 56 genes that clustered together with *Arabidopsis* type II genes were labeled as type II (Containing MIKC^c^ and MIKC^∗^clades). The ML tree had a consistent classification result (Fig. 1). The number of type II *MADS-box* genes was similar to those in *Arabidopsis* (55), rice (43), and poplar (64) (Parenicova et al., 2003; Arora et al., 2007; Leseberg et al., 2006). The number of type I genes was comparable to rice (32) and poplar (41) (Arora et al., 2007; Leseberg et al., 2006). Pear and apple, both members of Rosaceae, had the closest genetic relationship. To compare their gene numbers, we used the same identification method from pear to identify *MADS-box* genes in apple. A total of 142 *MADS-box* genes were found in apple, as in a recent report by Kumar et al. (2016). This demonstrated the reliability of the approach used to identify the *PbrMADS* genes. However, the number of *MADS-box* genes in apple were significantly more than in pear, suggesting that the *MADS-box* genes in pear underwent less gene duplication events or lost more repetitive genes than apple after their separation at 5.4–21 MYA (Million years ago) (Wu et al., 2013a). Assembled genome quality also led to gene number differences. The pear genome was assembled using a BAC-by-BAC approach, resolving problems of high heterozygosity and giving a high quality assembly and gene annotation. In contrast, the apple genome was sequenced using a WGS approach, which might lead to overestimation of gene numbers, due to alleles being annotated as different genes, as demonstrated by our previous genome research of pear (Wu et al., 2013a).

As conserved domains, *MADS* and K were easy to detect. Generally, type II proteins include both, while type I proteins only have the MADS domain. Based on SMART and NCBI CDD analysis, we found that 62 *PbrMADS* proteins only had MADS domains, while 33 had both MADS and K domains. Interestingly, 23 proteins lacking the K domain, similar to type I genes (Marked in Table 1), were classified as type II. A similar phenomenon was observed in rice *MADS-box* proteins (e.g., *OsMADS59*, *OsMADS37*, and *OsMADS65*) (Arora et al., 2007), and 28 non-K domain proteins could be also observed in apple type II genes (Tian et al., 2015). Here, five of 23 non-K domain genes were in the MIKC^∗^subfamily and the other 18 non-K domain genes were from 6 different subfamilies of MIKC^c^.

**Figure 2:**
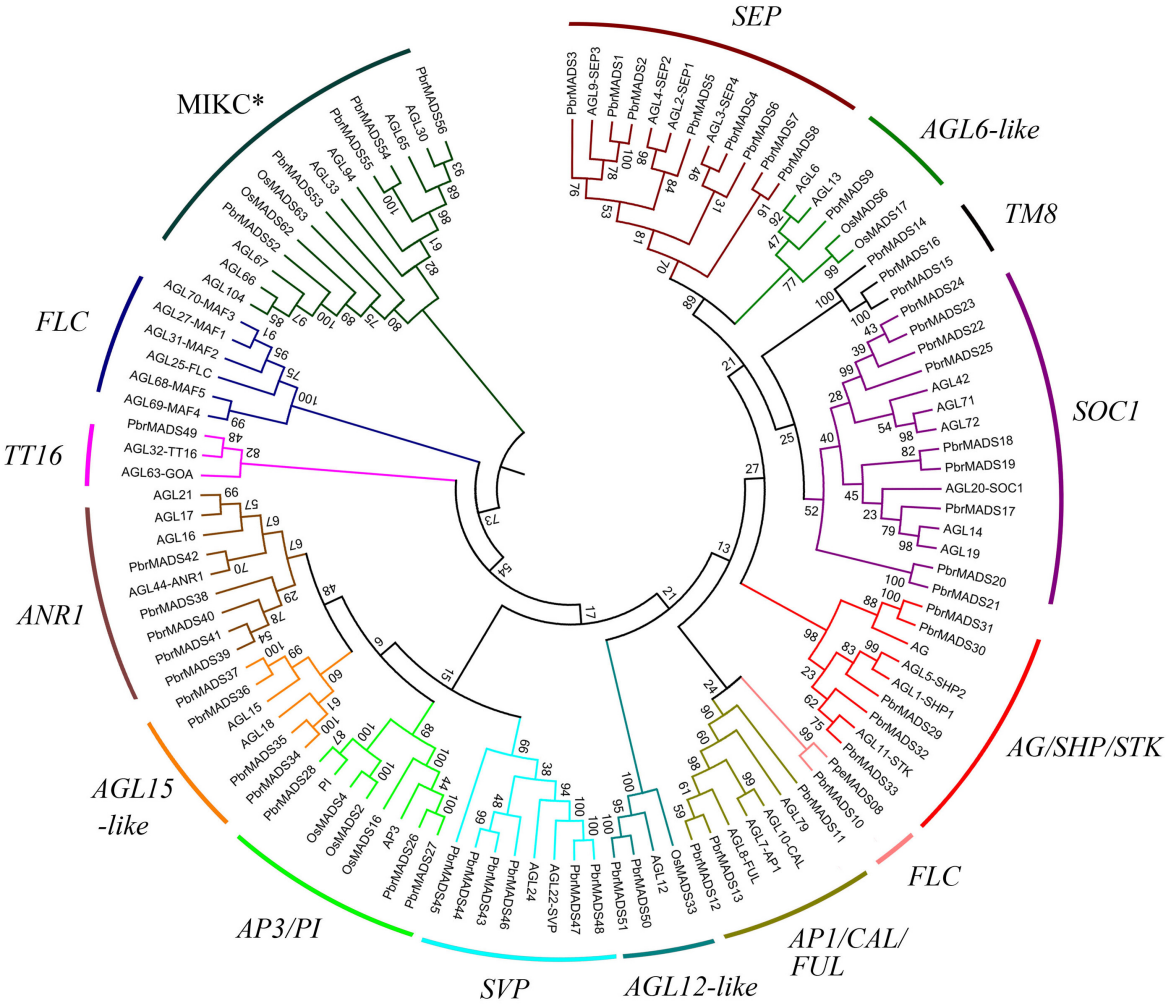
Phylogenetic tree of type II *MADS-box* transcription factors in pear, *Arabidopsis*, and rice. A total of 56 type II *MADS-box* proteins in pear, 46 in *Arabidopsis*, and eight in rice were used to construct the NJ tree. The subgroups are indicated by different branch colors.

In order to examine phylogenetic relationships of *MADS-box* genes in pear and classify them into different groups, two phylogenetic trees for type I and type II genes were constructed independently using *MADS-box* proteins of pear, *Arabidopsis*, and rice by the neighbor-joining method (Fig. 2 and Fig. S1). Furthermore, we used the ML method to confirm the results from the NJ method (Fig. S2). The topologies of the trees generated by the two methods were similar, indicating a reliable tree structure. Although some subgroups, such as *SOC1* and *SVP,* showed low bootstrap supports, this might be associated with the loss of K domain leading to large sequence divergence in same subgroup.

According to the phylogenetic trees, the pear type I *MADS-box* genes could be divided into three subfamilies, Mα (14 members), Mβ (seven members), and Mγ (18 members). Type II *MADS-box* genes were divided into 14 subfamilies, a similar result to *Arabidopsis* (Parenicova et al., 2003). Eleven subfamilies of the 14 had *Arabidopsis* counterparts. One subfamily was found to contain only pear members, including *PbrMADS14*, *PbrMADS15*, and *PbrMADS16*. To investigate their function, homology BLASTP searches were performed using the three protein sequences against the NCBI non-redundant protein database. These three proteins showed high identities of 75%, 100%, and 92% with *TM8* (*TOMATO MADS-box 8*)*-like* protein of *P. pyrifolia,* suggesting that they were *TM8* function proteins. No *TM8* genes have been reported in *Arabidopsis* (Becker & Theißen, 2003; Greco et al., 2011), but have been identified in tomato, grapevine, and poplar (Pnueli et al., 1991; Díaz-Riquelme et al., 2009). The *FLC* subfamily possessed six *Arabidopsis* genes, which have been implicated in the control of flowering via vernalization and autonomous pathways (Sheldon et al., 2000; Arora et al., 2007). This subfamily has been found in dicots, e.g., *Arabidopsis*, Chinese cabbage, grapevine, and from Rosaceae, apple and peach (Parenicova et al., 2003; Duan et al., 2015; Díaz-Riquelme et al., 2009; Porto et al., 2015; Wells et al., 2015). Two *FLC* genes were also found in monocot rice in a recent report (Ruelens et al., 2013). In our study, only one pear *MADS-box* gene was found in *FLC* subfamily and might play vital role for pear vernalization in flowering. Subfamilies *FLC*, *TT16*, and *AGL6-like* contained the minimum number (only one) of pear type II proteins, while *SOC1* subfamily contained the maximum number (up to nine). In *AGL12-like* and *AGL15-like*, each *Arabidopsis* gene had two orthologous genes from pear, indicating that additional lineage-specific duplication events in *Arabidopsis* or loss events occurred in pear for these two subfamily genes after the divergence of two the species.

The phylogenetic analysis of pear *MADS-box* genes is essential for comparative genomics research. In this study, subfamily classification allowed identification of the putative functions of *PbrMADS* genes. Pear and apple, both members of Rosaceae, had the closest genetic relationship. Currently, the most extensive functional research has been done in *MADS-box* gene of apple. Genes in the same subfamilies for both apple and pear could provide a reference for gene function. For instance, *SEPALLATA1*/*2*-like genes were reported to control fruit flesh development and ripening (Ireland et al., 2013). Transposon insertion mutants of *MdPI* are responsible for the flower and fruiting phenotype of apple mutants (Yao, Dong & Morris, 2001). Transgenic suppression of *AGAMOUS* genes in apple reduces fertility and increases floral attractiveness (Klocko et al., 2016). In addition, the functions of apple *MADS-box* genes in *AP1* and *SHP* subfamily have also been characterized (Yao et al., 1999; Van der Linden, Vosman & Smulders, 2002). These results provide hypothetical gene functions for *MADS* genes in pear involving in fruit and flower development.

### Gene structure and conserved motif analysis of the *MADS-box* genes

**Figure 3:**
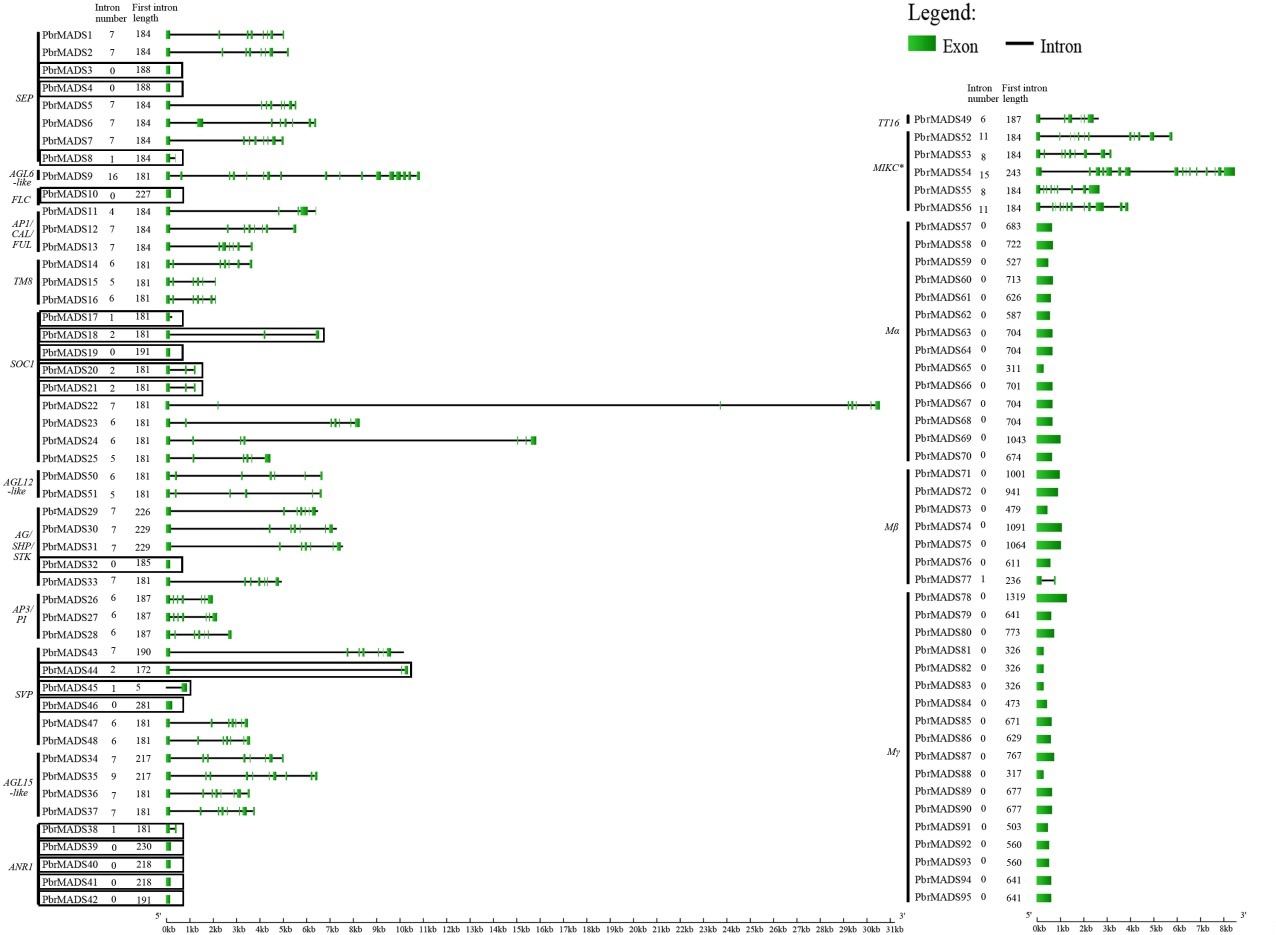
Gene structure analysis of *MADS-box* transcription factors in pear. Exons are represented by green boxes, and introns by black lines. Each gene is shown proportionally using lengths of exons and introns. Non-K domain genes are marked with black boxes. Intron number and first exon length of each gene are indicated following gene name.

To understand the structural diversity of *MADS-box* genes in pear, intron-exon organization was analyzed (Fig. 3). Like *Arabidopsis* and rice, a prominent bimodal distribution of introns could also be observed in pear type I and type II genes, and MIKC^∗^ genes contained more introns compared with MIKC^c^ genes (Parenicova et al., 2003; Arora et al., 2007; Hu & Liu, 2012). Eighteen MIKC^c^ non-K domain genes seemed to be inconsistent with other members because of low intron numbers ranging from 0 to 2 (Fig. 3). However, report has shown that MIKC^c^ genes are conserved in the lengths of first six exons (Johansen et al., 2002). By investigating the first exon length, we found that the 18 non-K domain genes (183 bp) were highly similar to others in type II (188 bp), less than the average length of type I genes (658 bp) (Fig. 3). Therefore, these 18 non-K domain genes were type II. This result further proved the reliability of pear *MADS-box* protein pre-classification.

The MEME program was then employed to analyze conserved motifs of pear *MADS-box* proteins. To better observe original motif distributions of different subfamilies, a conserved motif figure was made (Fig. S3) and the 23 non-K domain *MADS-box* genes were combined to show the different protein structures. A total of 20 conserved motifs, named 1 to 20, were identified (Table S2). Motif 1 and motif 2 represent the MADS domain. All type II and Mα proteins contained motif 1 except for *PbrMADS44, 45, 62*, and *69*. Most Mβ and Mγ proteins had motif 2. Motifs 4, 7, and 9 were three fragments of the K domain. Apart from 23 non-K domain genes, other type II genes contained 1–3 members of motifs 4, 7, and 9. As shown in Fig. S3, pear type II *MADS-box* proteins were found to possess similar structure for every subfamily, whereas type I proteins showed more motif variation beyond the conserved MADS domain. Some specific motifs were particular to specific subfamilies, for example, motif 5 for Mγ subfamily, motif 15 for MIKC^∗^ subfamily, motif 18 for *SOC1* subfamily and motif 19 for *TM8-like* subfamily. Specific motifs may be the main cause of functional diversification between different subfamilies. The 23 non-K domain genes, except for *PbrMADS44* and *45*, had a similar type of MADS domain as other members in type II. However, when observing the C-terminal regions, 5 genes belonging to MIKC^∗^ showed big differences in motifs with other type II genes and 18 genes of the MIKC^c^ type seemed to have lost some motifs. These differences might have derived from the evolution of *MADS* genes in pear. Gene duplication prior to the divergence of plants and animals may have given rise to the two main lineages of *MADS-box* genes: types I and II (Alvarez-Buylla et al., 2000), and supported by another report (Nam, Ma & Nei, 2003). A gene duplication event occurred in type II genes after land plant origin, leading to MIKC^c^ and MIKC^∗^ proteins (Henschel et al., 2002). We speculated that five MIKC^∗^ non-K domain pear genes or their ancestral genes underwent structural divergence in C-terminal regions, while 18 non-K domain genes or their ancestral genes in MIKC^c^ experienced large fragment loss, which both resulted in non-K domain type II genes.

**Figure 4:**
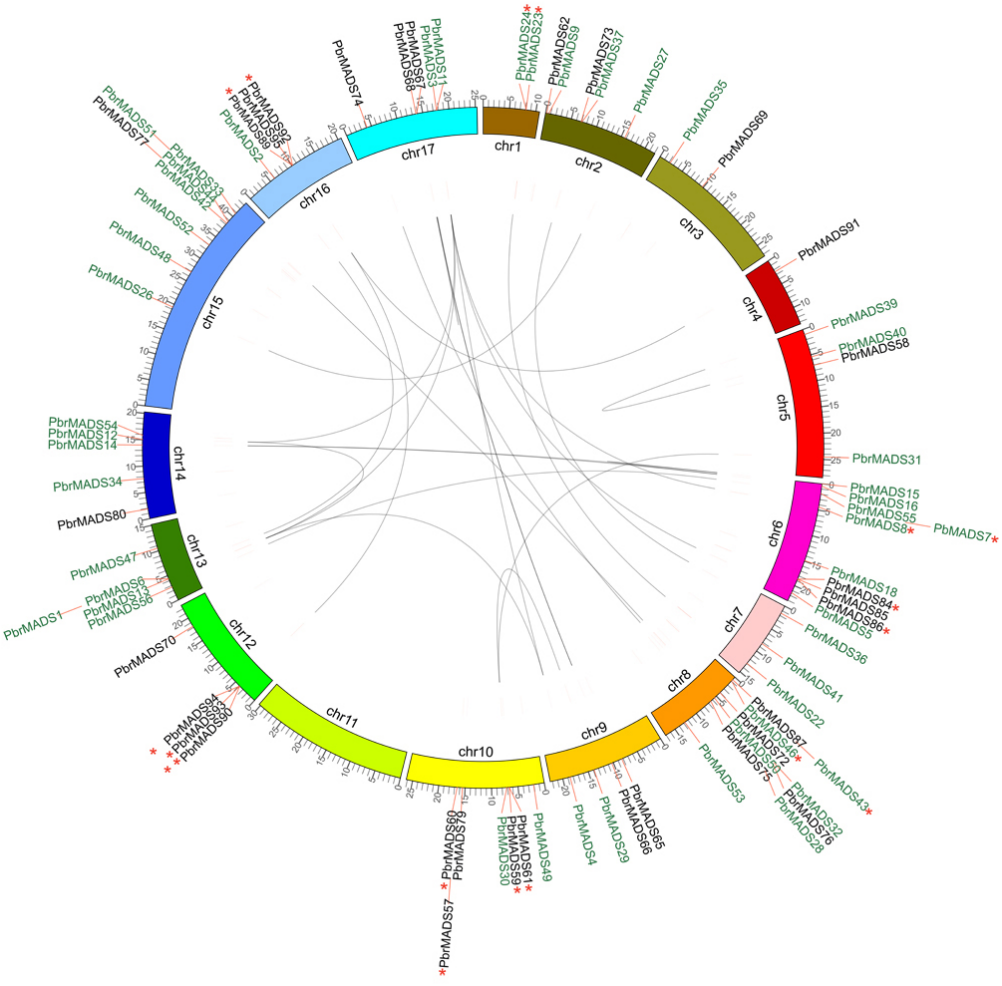
Chromosomal location and synteny relationship of the *MADS-box* genes in pear. A total of 17 chromosomes of pear marked by different colors and labeled with their names, chr1 to chr17, on the inner side. Different types of *MADS-box* genes are denoted by different colors: type I black and type II green. WGD or segmental duplication gene pairs are joined by black lines. Tandem duplication gene pairs are marked by red stars.

### Chromosomal locations and expansion of the *PbrMADS* gene family revealed by synteny analysis

According to genome annotation files, 79 of 95 *MADS-box* genes were located on pear chromosomes, while 16 of them were on the scaffolds. The *MADS-box* genes showed uneven distribution on pear 17 chromosomes. As shown in Fig. 4, chromosome 11 did not contain *MADS-box* genes, while chromosomes 6 and 8 had the highest numbers of *MADS-box* genes, up to 10. Most genes were clustered on certain regions of the chromosome, instead being evenly distributed, possibly from uneven duplication events of chromosome fragments (Wu et al., 2013a). Gene duplication is one of the prevalent forces resulting in increased gene numbers and genome complexity in eukaryotes (Li et al., 2001; Hughes, 1994; Kaul et al., 2000). It is estimated that genome duplication has been directly responsible for more than 90% of the increase of regulatory genes in the *Arabidopsis* lineage (Maere et al., 2005). Gene duplication modes—WGD (Whole-genome duplication) or segmental duplication, tandem duplication, and rearrangement events—are the main drivers of evolution of gene families (Kong et al., 2007). We used MCScanX to detect gene duplication in the *MADS-box* transcription factor family in pear and *Arabidopsis*, and found 37 segmental duplication genes (25 WGD or segmental duplication events), 17 tandem duplication genes (9 tandem duplication events), and 35 dispersed genes in pear. The corresponding numbers were 50, 11, and 24 in *Arabidopsis.* The results showed that expansion mechanisms of *MADS-box* transcription factor family were different between pear and *Arabidopsis.* For *Arabidopsis*, WGD or segmental events play a more important role, while WGD or segmental and rearrangement events were more prevalent in the *PbrMADS* gene family, indicating their critical roles in the expansion of the *MADS* family.

**Figure 5:**
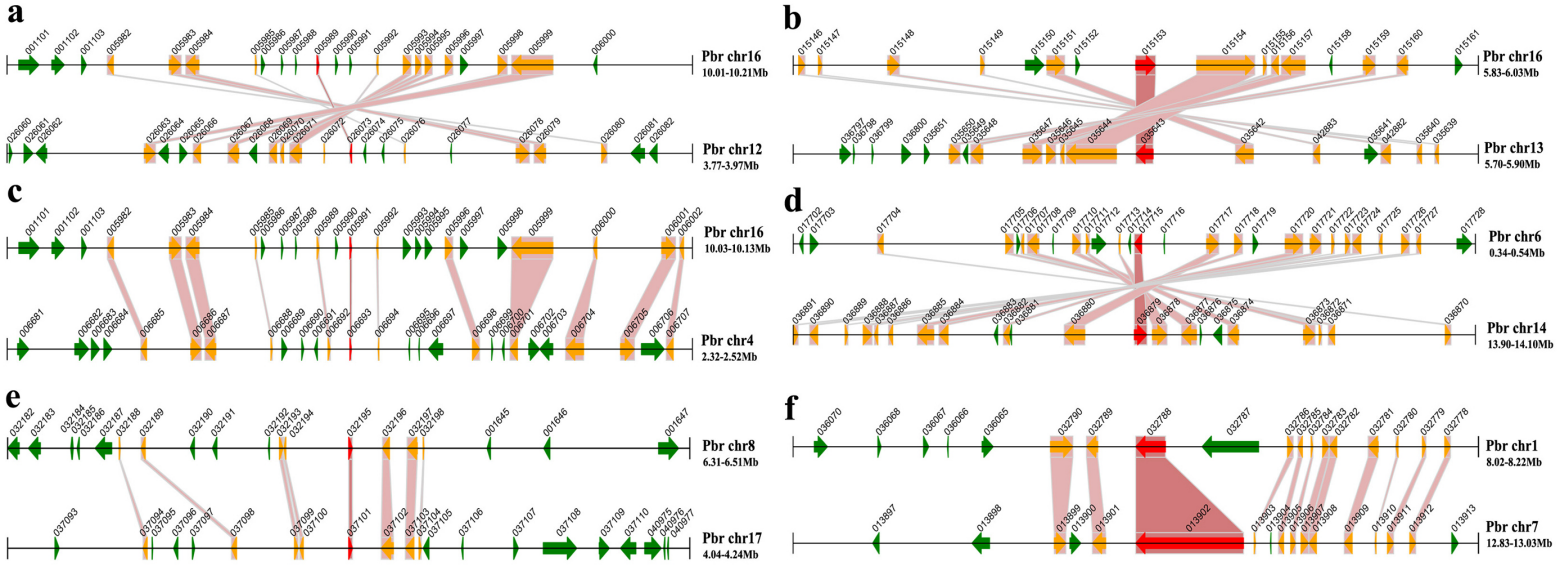
Segmental duplication of the *MADS-box* family in pear. A region of 100 kb flanking each side of the *MADS* gene is displayed. The black horizontal line denotes a chromosome segment with the chromosome name and region on the right, and the gene and its transcription orientation is indicated by a broad line with an arrowhead. The text beside the line is the gene name suffix. The *MADS* genes are shown in red, homologous genes in yellow, and other genes in green. Homologous gene pairs are linked with red bands. (A) *PbrMADS95* (*Pbr005989.1*) and *PbrMADS93* (*Pbr026073.1*) (B) *PbrMADS2* (*Pbr015153.1*) and *PbrMADS1* (*Pbr035643*.1) (C) *PbrMADS92* (*Pbr005991*.1) and *PbrMADS91* (*Pbr006693*.1) (D) *PbrMADS16* (*Pbr017715*.1) and *PbrMADS14* (*Pbr036879*.1) (E) *PbrMADS75* (*Pbr032195.1*) and *PbrMADS74* (*Pbr037101*.1) (F) *PbrMADS23* (*Pbr032788*.1) and *PbrMADS22* (*Pbr013902.1*).

To further investigate the potential evolutionary mechanisms of the *PbrMADS* gene family, a method similar to that developed for the PGDD (Plant Genome Duplication Database) was used to identify synteny blocks across the pear genome. All 34 conserved synteny blocks, including 25 segmental *MADS-box* gene pairs, were observed across the pear genome (Fig. 5 and Table 2). Among 25 segmental *MADS-box* gene pairs, 19 belonged to type II and six to type I, which might contribute to the greater gene numbers in type II than type I. In order to prove that segmental duplications were real, we searched the genes and homologous gene pairs in 100 kb flanking each side of the 25 segmental *MADS-box* gene pairs, and found many genes within flanking region from segmental duplication. The number of genes and homologous gene pairs found were up to 53 and 18, respectively, within the 200 kb window among different synteny blocks. These results further demonstrated the occurrence of WGD or segmental duplication, leading to the expansion of the *MADS-box* gene family in pear.

**Table 2:** Synteny analysis of *MADS-box* gene regions in pear genome.

Duplicated MADS-box gene 1	Duplicated MADS-box gene 2	Gene type	Mean Ks	Homologous gene pairs in 200 kb	Genes in 200 kb
PbrMADS39	PbrMADS40	Type II	0.04	7	23
PbrMADS15	PbrMADS16	Type II	0.04	13	30
PbrMADS68	PbrMADS67	Type I	0.05	10	28
PbrMADS74	PbrMADS75	Type I	0.09	8	41
PbrMADS3	PbrMADS5	Type II	0.09	6	38
PbrMADS68	PbrMADS66	Type I	0.17	3	28
PbrMADS3	PbrMADS4	Type II	0.18	6	44
PbrMADS30	PbrMADS31	Type II	0.18	8	46
PbrMADS51	PbrMADS50	Type II	0.19	7	38
PbrMADS37	PbrMADS36	Type II	0.20	8	48
PbrMADS1	PbrMADS2	Type II	0.20	12	35
PbrMADS14	PbrMADS15	Type II	0.21	8	37
PbrMADS14	PbrMADS16	Type II	0.23	18	44
PbrMADS26	PbrMADS27	Type II	0.24	7	22
PbrMADS93	PbrMADS95	Type I	0.24	12	45
PbrMADS23	PbrMADS22	Type II	0.25	12	35
PbrMADS92	PbrMADS91	Type I	0.25	12	48
PbrMADS67	PbrMADS66	Type I	0.29	3	30
PbrMADS3	PbrMADS8	Type II	1.06	4	44
PbrMADS6	PbrMADS4	Type II	1.33	6	53
PbrMADS30	PbrMADS29	Type II	1.56	2	40
PbrMADS56	PbrMADS55	Type II	1.75	5	50
PbrMADS12	PbrMADS11	Type II	1.77	2	42
PbrMADS13	PbrMADS11	Type II	2.22	2	49
PbrMADS13	PbrMADS29	Type II	2.99	2	46

**Notes.**

Homologous gene pairs in the 100 kb flanking each side of the *PbrMADS* genes were chosen to estimate the mean Ks. The number of genes in 200 kb was a total number of two segments.

### History of duplication events and driving forces for evolution of the *MADS-box* family

The Ks value (synonymous substitutions per site) is widely used as a proxy for time to calculate approximate dates of WGD or segmental duplication events. Wu et al. (2013a) stated that two genome-wide duplication events took place in the pear genome: an ancient WGD (Ks ∼1.5–1.8) derived from a paleohexaploidization (γ) event around 140 MYA (Fawcett, Maere & Peer, 2009), and a recent WGD (Ks ∼0.15–0.3), inferred to have originated 30 to 45 MYA. Therefore, we used Ks value to trace the date of segmental duplication events within the *PbrMADS* transcription family. The mean Ks values of the *PbrMADS* duplicated gene pairs in the syntenic region are shown in Table 2, and ranged from 0.04 to 2.99. The segmental duplications *PbrMADS29* vs. *PbrMADS30* (Ks ∼1.56), *PbrMADS55* vs. *PbrMADS56* (Ks ∼1.75), and *PbrMADS11* vs. *PbrMADS12* (Ks ∼1.77) might have resulted fromγ triplication (∼140 MYA), because their Ks values were within the Ks scope of the ancient WGD in pear. Moreover, Ks values of 13 duplicated gene pairs were 0.17–0.29, suggesting that these duplications might have arisen from the same recent WGD (30∼45 MYA). Some gene pairs were not distributed on either of the two WGD events. Two duplicated gene pairs (*PbrMADS11* vs. *PbrMADS13* and *PbrMADS13* vs. *PbrMADS29*) with higher Ks values (2.22–2.99) probably originated from a more ancient duplication event. In addition, five duplicated gene pairs (*PbrMADS39* vs. *PbrMADS40*, *PbrMADS15* vs. *PbrMADS16*, *PbrMADS67* vs. *PbrMADS68*, *PbrMADS74* vs. *PbrMADS75* and *PbrMADS3* vs. *PbrMADS5*) had lower Ks values of 0.04-0.09, and two duplicated gene pairs (*PbrMADS3* vs. *PbrMADS8* and *PbrMADS4* vs. *PbrMADS6*) had Ks values of 1.06 and 1.33. On the one hand, these results could indicate a more recent duplication event and the period between the recent and ancient WGDs, respectively. On the other hand, their values might reflect deviations affected by gene conversion events and might have resulted from the recent and ancient WGDs. Concerted evolution via gene conversion is recognized as a major feature in the evolution of multigene families (Michelson & Orkin, 1983; Aguileta, Bielawski & Yang, 2004; Teshima & Innan, 2004). Gene conversion, one of the two mechanisms of homologous recombination, can be functionally defined as the nonreciprocal transfer of material from one region of DNA to another (Goldstone & Stegeman, 2006). Segmentally duplicated sequences showed high similarity through gene conversion, thus causing lower Ks rates. In our study, mean Ks values of duplicated gene pairs in the syntenic region were used to reduce the deviation.

In the study of molecular evolution, a basic issue is the distinction between adaptive, neutral, and deleterious mutations (Fay, Wyckoff & Wu, 2001). Although adaptive mutation, and their maintenance are considered the key to Darwinian evolution, most of the accumulated DNA changes are likely to be neutral, maintained randomly in a population (Kimura, 1983). However, there is evidence of the existence of adaptive evolution for some proteins (Nei, 1987), leading to functional divergence (Starr, Jameson & Hogquist, 2003). On the other hand, negative selection reduces the ratio of amino acids to synonymous divergence between populations, and the proportion of deleterious amino acid-altering mutations can be estimated using this ratio (Fay, Wyckoff & Wu, 2001). Demonstration that a protein has evolved more rapidly than the neutral substitution rate requires a comparison of the number of non-synonymous substitutions per non-synonymous site (termed Ka), with the number of synonymous substitutions per synonymous site (termed Ks) between homologous gene pairs (Li, Wu & Luo, 1985). A Ka/Ks ratio of 1 indicates neutral selection, <1 indicates negative selection, and >1 indicates positive selection (Yang & Nielsen, 2000). To investigate what kind of selection pressure drove the evolution of the *MADS* gene family in pear, we calculated the nonsynonymous/synonymous substitution (Ka/Ks) ratios for the full-length coding regions of segmental and tandem duplicated gene pairs (Table S3). A boxplot result showed the Ka/Ks ratio of duplicated genes for *MADS* genes of pear vs. the other genes of pear and *MADS* genes of apple (Fig. S4). Duplicated genes in pear had a mean Ka/Ks value of 0.34. The mean Ka/Ks values of tandem and segmental duplicated genes in other pear genes and apple *MADS* genes were 0.57 and 0.51. The confidence intervals of all were less than 1. An independent sample Mann–Whitney *U*-test showed that Ka/Ks ratios of *MADS* genes in pear were significantly higher than the other genes of pear and *MADS* genes of apple (both *p*-values equal 0), demonstrating that pear *MADS* genes showed a low evolutionary rate and experienced strong purifying selective pressure. We deduced that purifying selection might contribute to the maintenance of *MADS* gene function in pear. *MADS* gene pairs resulting from tandem duplication have a low Ka/Ks ratio with an average of 0.448, ranging from 0.179 to 0.645. Segmental duplicated MADSs also have a low Ka/Ks ratio with an average of 0.290, ranging from 0.035 to 0.794. Ka/Ks ratio of tandem duplicated *MADS* genes was significantly higher than segmental duplicated *MADS* genes according to the Mann–Whitney *U*-test, indicating that tandem duplicated *MADS* genes experienced a lower evolutionary rate than segmental duplicated *MADS* genes. These observations indicated that duplicated MADSs have primarily experienced purifying selective pressure.

Previous research has proven the expansion of positive selection on many protein families via phylogeny-based analyses of codon substitution (Smith & Eyre-Walker, 2002; Yang, Wong & Nielsen, 2005) and positive selection at some codons was an important driving force for protein evolution (Yang & Nielsen, 2002). To further detect whether Darwinian positive selection was involved in a few amino acid residues of *PbrMADS* proteins, the branch-site model method was used to calculate ML estimation of the Ka/Ks substitution rate ratios for 34 gene pairs, in which each sequence came from the same duplication event at nodes in the pear and apple *MADS* protein phylogeny (Fig. S5, Table S4). In this study, 17 gene pairs were under positive selection for foreground lineages Prob (*ω* > 1) according to Bayes Empirical Bayes (BEB) analysis. Among them, one gene pair of the *SVP* subfamily (*PbrMADS43* and *PbrMADS46*) could be detected three positive codon sites (FDR=0.0306), indicating a functional divergence. The *SVP* subclade has been implicated with the regulatory function of floral transition and bud dormancy (Hartmann et al., 2000; Wu et al., 2012), so these two *MADS* genes might also play roles in these processes.

### Expression analysis of pear *MADS-box* transcription factor family

Firstly, pear *MADS-box* genes were submitted to EST database to verify the accuracy of the previous genomic predictions. The results provided reliable transcriptional evidence for most of these *PbrMADS* genes: of the 95 *MADS-box* genes, 56 were found to have EST hits (Table 1). However, no EST hits were identified for 39 *MADS* genes. Of these 39 genes, five were from *ANR1* subgroup, two from *AGL12*-like subgroup, and 12, 6, and 14 from M α, Mβ, and Mγ clades, respectively. The expressions of 39 genes were further investigated using previously published transcription data of fruit development and pollen in pear (*P. bretschneideri*) (Zhou et al., 2016; Wu et al., 2013a). Indeed, 25 of them were found without expression in pear fruit and pollen, and the others showed little expression either in one of the two tissues or both tissues (Table S5). These genes were either pseudogenes or only expressed under certain conditions, in specific cell types, or at limited developmental stages. The functional roles of these genes will require further investigation in specific study.

**Figure 6:**
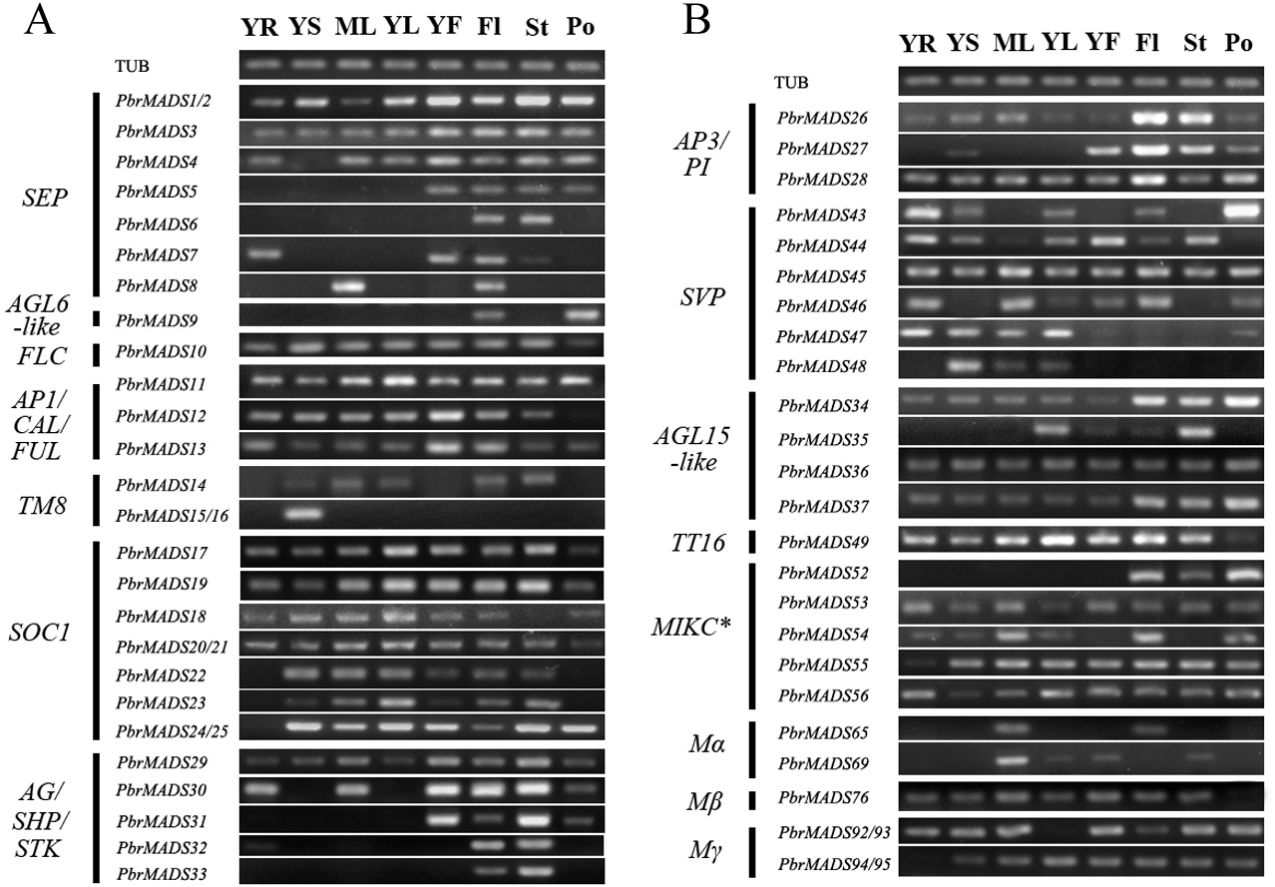
Expression patterns of pear *MADS-box* genes in vegetative and reproductive organs. (A) The first part. (B) The second part. Total RNA was isolated from young root (YR), young stem (YS), mature leaf (ML), young leaf (YL), young fruit (YF), flower (Fl), style (St), and pollen (Po). *Pyrus* TUB was used to adjust cDNA concentration and it is for both (A) and (B).

To further survey the expression patterns of 56 *PbrMADS* genes having reliable transcriptional support, RT-PCR was carried out in vegetative and reproductive organs, including young root, young stem, mature leaf, young leaf, young fruit, flower, style, and pollen (Fig. 6). Many *MADS-box* genes showed wide expression spectrums: a total 38 of 56 genes (68%) were expressed in at least six of the eight tested tissues, indicating that *MADS* genes have extensive functions in different tissues of pear. A total of 54 of 56 (96%) *PbrMADS* genes were expressed in flower, demonstrating the vital function of *MADS-box* genes for flowering. Some *MADS* genes were expressed in a specific type of tissue, for example, the expression of *PbrMADS5* was restricted to reproductive organs and *PbrMADS48* expression was detected specifically in vegetative tissue. *SEP* genes acted as the E function genes required for floral organ identity (Pelaz et al., 2000; Ditta et al., 2004; Ferrario et al., 2003). The *PbrMADS5* belonging to *SEP* may have a similar role in flower, and this putative function was supported by its specific expression in reproductive organs. *SVP* subclade has been implicated in the regulations of floral transition in *Arabidopsis* (Hartmann et al., 2000), and four *SVP* genes in kiwifruit also played a role in bud dormancy, their expression was generally confined to vegetative tissues (Wu et al., 2012). For *PbrMADS48*, a member of *SVP* subclade, more attention should be focused on the function of bud dormancy rather than floral transition, because of the specific expression in vegetative tissue. Moreover, five *MADS* genes were found to have an expression signal in specific tissues, e.g., *PbrMADS6* and *33* from *SEP* and *AG/SHP/STK* subfamilies in style, *PbrMADS9* from *AGL6-like* in pollen, *PbrMADS15* and *16* from *TM8-like* in stem, and *PbrMADS52* from MIKC^∗^ in flower, indicating crucial roles in the development of these tissues. *PbrMADS6*, a member *SEP* subclade, expressed in style, suggesting its specific function for style identity. *STK* regulated ovule identity and could promote carpel development (Angenent et al., 1995; Dreni et al., 2007; Favaro et al., 2003)*. PbrMADS33*, a homolog of *Arabidopsis STK* gene, suggested a unique function in style development by its specific expression. *AGL6-like* subclade genes are involved in regulations of floral organ and meristem identities (Ohmori et al., 2009; Li et al., 2010; Rijpkema et al., 2009), for example *PbrMADS9*, which may demonstrate its vital function in pollen identity. *TM8* was isolated from the floral meristem of tomato more than twenty years ago (Pnueli et al., 1991), but its function is still poorly known. Recently, it was suggested that the *TM8* protein played a role in development of the tomato flower (Daminato et al., 2014). *PbrMADS15* and *16* only expressed in stem, which suggested a potentially novel function. Generally, functions of MIKC^∗^ genes are less clear than MIKC^c^ genes. MIKC^∗^ genes were required for the pollen maturation in *Arabidopsis* and rice (Adamczyk & Fernandez, 2009; Liu et al., 2013a), so *PbrMADS52* might similarly play an important role in pear pollen maturation.

Paralogous genes generated by gene duplication in the same genome usually have similar functions (Zhang, 2003). To explore whether paralogous *MADS* genes in pear have parallel functions, we analyzed the expression patterns of 14 paralogous *MADS* gene pairs in 12 segmental- and 2 tandem-duplicated gene pairs. Another 20 duplicated gene pairs were disregarded for not having a transcriptional signal or gene-specific primer with their paralogous genes. The expression results showed that some gene pairs (*PbrMADS11* and *PbrMADS13*, *PbrMADS13* and *PbrMADS29*, *PbrMADS22* and *PbrMADS23*, *PbrMADS36* and *PbrMADS37*, and *PbrMADS55* and *PbrMADS56*) exhibited similar expression profiles, indicating the conserved functions of these gene pairs. Conversely, some paralogous *MADS* gene pairs (*PbrMADS3* and *PbrMADS4*, *PbrMADS3* and *PbrMADS5*, *PbrMADS3* and *PbrMADS8*, *PbrMADS4* and *PbrMADS6*, *PbrMADS7* and *PbrMADS8*, *PbrMADS11* and *PbrMADS12*, *PbrMADS26* and *PbrMADS27*, *PbrMADS30* and *PbrMADS31*, and *PbrMADS43* and *PbrMADS46*) were found to have different expression patterns, indicating functional divergence of these gene pairs after duplication. Five of these pairs were from *SEP* subfamily, showing its large variation. The different expression patterns of the segmental gene pair *PbrMADS43* and *PbrMADS46*, accompanied with many positive selection sites, strongly indicated functional divergence of these two genes. The results suggested that gene duplication events played critical roles in gene family evolution, because duplicated genes are major contributors to the raw materials for the emergence of new functions through the forces of mutation and natural selection (Kong et al., 2007).

In general, gene expression patterns in same functional clade were conserved. Three genes in the *AP1/CAL/FUL* subgroup were widely expressed in tested tissues, except for *PbrMADS12*, which has no expression in pollen. However, members in same group may also exhibit diverse expression patterns. In *AG/SHP/STK/* subgroup, *PbrMADS29* expressed in all analyzed tissues, *PbrMADS30* in most tissues except for stem and young leaf, *PbrMADS31* in reproductive organs, *PbrMADS32* in root and style, and *PbrMADS33* in style. The expression profiles of *PbrMADS* genes also show large differences at different developmental stages of tissues: *PbrMADS8*, *PbrMADS30*, *PbrMADS65*, and *PbrMADS92/93* were expressed in mature leaves, while *PbrMADS35* and *PbrMADS43* were observed in young leaves. Interestingly, expression of *PbrMADS47* and *PbrMADS69* were detected in pollen and style separately, but cannot be detected in the entire flower. It could be that the expression signal was too low to be detected.

### The role of *PbrMADS11* and *PbrMADS12* on anthocyanin synthesis

Given that our work is focused on improving the quality of pear fruit, and fruit nutrients such as anthocyanin are one of the most important aspects of pear quality for consumers, we were interested in the role of *MADS-box* genes in fruit anthocyanin pathway. They have been reported to be involved in anthocyanin accumulation and regulation in previous work, but this function has been little studied in pear. Therefore, fruit skin of the red-colored ‘Starkrimson’ and its green variant strain at four different stages (at 40, 55, 70, and 85 days after full bloom, DAFB) was used as material to explore whether *PbrMADS* genes cause the color difference. We collected protein sequences reported to be involved in anthocyanin accumulation and regulation to construct a phylogenetic tree with identified pear *MADS-box* proteins (Table S6 and Fig. S6) (Jaakola et al., 2010; Lalusin et al., 2006; Nesi et al., 2002). Seven genes (*PbrMADS10*, *PbrMADS11*, *PbrMADS12*, *PbrMADS13*, *PbrMADS49*, *PbrMADS50*, and *PbrMADS51*) clustered in the same clade with anthocyanin related genes reported in other plants, which were considered candidates for anthocyanin accumulation and regulation in pear. They were in four different subfamilies. *PbrMADS11*, *PbrMADS12*, and *PbrMADS13* genes were in *AP1/CAL/FUL* subfamily, *PbrMADS10* in *FLC* subfamily, *PbrMADS49* in *TT16* subfamily, and *PbrMADS50* and *PbrMADS51* in *AGL12-like* subfamily.

**Figure 7:**
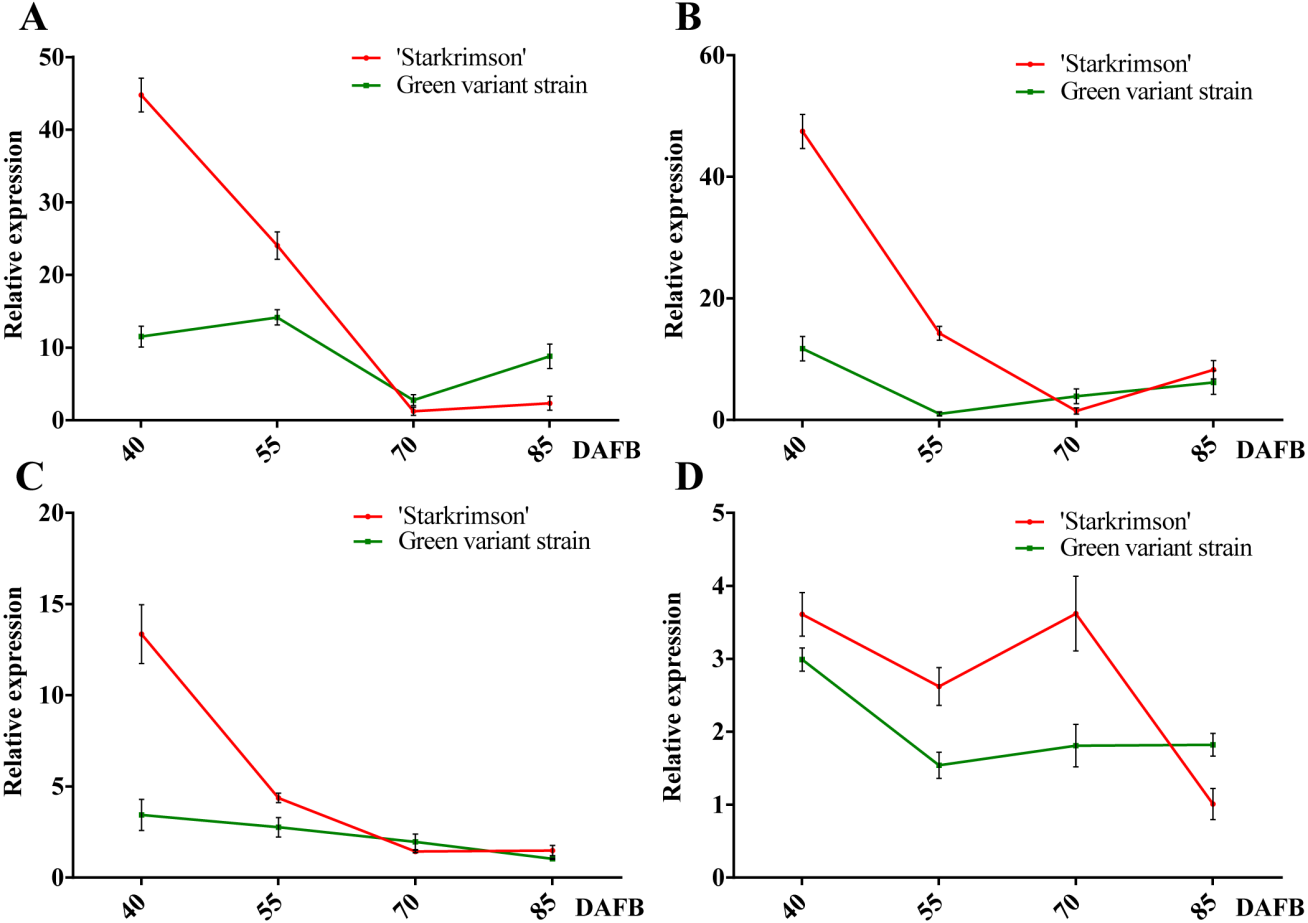
Relative expressions of four *PbrMADS* genes at different stages of fruit development in ‘Starkrimson’ and its green variant strain. (A) *PbrMADS10*, (B) *PbrMADS11*, (C) *PbrMADS12*, (D) *PbrMADS13*. Error bars indicate standard deviation for three replicates.

qRT-PCR was then used to verify the validity of candidate genes. Before qRT-PCR, a RT-PCR experiment was carried out using pooled cDNA samples to determine whether the seven genes are expressed in peel of red-colored ‘Starkrimson’ and its green variant strain. No target bands were found for *PbrMADS49*, *PbrMADS50*, and *PbrMADS51* (data not show), suggesting that they were not target genes related to pigmentation, and *PbrMADS10*, *PbrMADS11*, *PbrMADS12*, and *PbrMADS13* were used for further qRT-PCR analyses (Fig. 7). The relative expression level of *PbrMADS10* in red-skinned ‘Starkrimson’ was highest at the first stage of fruit development, decreased until 70 DAFB, and was then slightly upregulated near ripening. In the green variant strain, *PbrMADS10* presented a maximum expression level at 55 DAFB, and gradually declined until the later stage (70 DAFB), with a slight rise close to maturation. The expression levels of *PbrMADS11* and *PbrMADS12* show a similar tendency relative to *PbrMADS10* in red-skinned fruit. For green-skinned fruit, the expression of *PbrMADS11* dropped at 55 DAFB, and decreased until 85 DAFB, while *PbrMADS12* went down from 45 to 85 DAFB. *PbrMADS13* displayed a drop-rise-drop pattern in red pear, and displayed a drop-rise-rise profile in green pear. The expression levels of *PbrMADS10*, *PbrMADS11*, and *PbrMADS12* genes in red strains were 3.9-fold, 4.0-fold, and 3.9-fold higher than the green mutant strain at 40 DAFB, respectively. Except for the expression of *PbrMADS11* at 55 DAFB, expression levels in red skin fruit were 14.0-fold higher than green fruit, but no more than 2-fold more at later stages for the three genes. These three genes may have an important role in anthocyanin accumulation, especially in early phases of fruit development. We have previously reported the variation of anthocyanin content of ‘Starkrimson’ and green variant strain during four different developmental stages (40, 55, 70, and 85 DAFB) (Wu et al., 2013b). ‘Starkrimson’ exhibited a drop-rise-drop pattern, with the highest values at 40 DAFB and the green variant strain showed a drop-rise-drop pattern, varying steadily at a low level (Fig. S7). Through correlation analysis, a positive correlation was found between the variation of anthocyanin content and expression of *PbrMADS11* and *PbrMADS12* in the red-skinned ‘Starkrimson’, indicating an important relationship between anthocyanin content and gene expression (Table S7). These data indicated that *PbrMADS11* and *PbrMADS12* were important candidate genes in the regulation of anthocyanin biosynthesis, and mainly function in the early period of fruit development.

Anthocyanin biosynthesis has been reported to be controlled by transcriptional regulators of the *MYB*, *bHLH* and *WD40* genes (Grotewold, 2006). These regulators can activate promoters of the anthocyanin biosynthetic genes by forming a MBW complex (Gonzalez et al., 2008). Besides the MBW complex, regulatory genes and transcription factors such as WRKY (Johnson, Kolevski & Smyth, 2002), *MADS* (Nesi et al., 2002), PIF3 (Shin, Park & Choi, 2007), NAC (Morishita et al., 2009) and COP1 (Maier et al., 2013) have also been reported to be involved in anthocyanin synthesis of *Arabidopsis*. The relationship of *MADS-box* genes with anthocyanin accumulation have been reported in different species. In *Arabidopsis*, *TT16* (*TRANSPARENT TESTA 16*) gene, encoding *ABS* (*ARABIDOPSIS B-SISTER*) *MADS-box* protein, is needed for seed coat pigmentation (Nesi et al., 2002). *IbMADS10* and *VmTDR4* genes take part in anthocyanin accumulation in sweet potato (*Ipomoea batatas*) and bilberry (*Vaccinium myrtillus*), respectively (Jaakola et al., 2010; Lalusin et al., 2006). *PyMADS18* was found likely to be involved in anthocyanin accumulation and regulation in early pear fruit development stage (Wu et al., 2013b). Over-expression of the *SVP3* gene in kiwifruit (*Actinidia spp.*) restricts anthocyanin biosynthesis in petals (Wu et al., 2014). A *MADS-box* transcription factor, *VmTDR4*, from bilberry was hypothesized to control anthocyanin accumulation (Jaakola et al., 2010). However, the nature of the interaction between *MADS-box* transcription factor and anthocyanin accumulation is unclear (Laura, 2013). Our previous research isolated seven anthocyanin biosynthesis genes (*PAL*, *CHS*, *CHI*, *DFR*, *F3H*, *ANS*, and *UFGT*) from ‘Starkrimson’ and the green variant strain, and proved that they were the main structural genes in the anthocyanin synthesis pathway of pear (Yang et al., 2013). The expression pattern of seven structural genes and three transcription factors of *PbMYB10*, *PbbHLH3* and *PbWd40* were detected in our previous study (Yang et al., 2013). The results showed that most of structural genes in anthocyanin synthesis pathway were up-regulated in the red-skinned ‘Starkrimson’ during fruit development, except for the *CHI* and *UFGT* genes were highly expressed only at an early stage. The expression levels of *PbMYB10* gene in the ‘Starkrimson’ were significantly higher than green mutant at the early stage, while the expression levels of *PbbHLH* and *PbWD40* were higher at a later stage.

**Figure 8:**
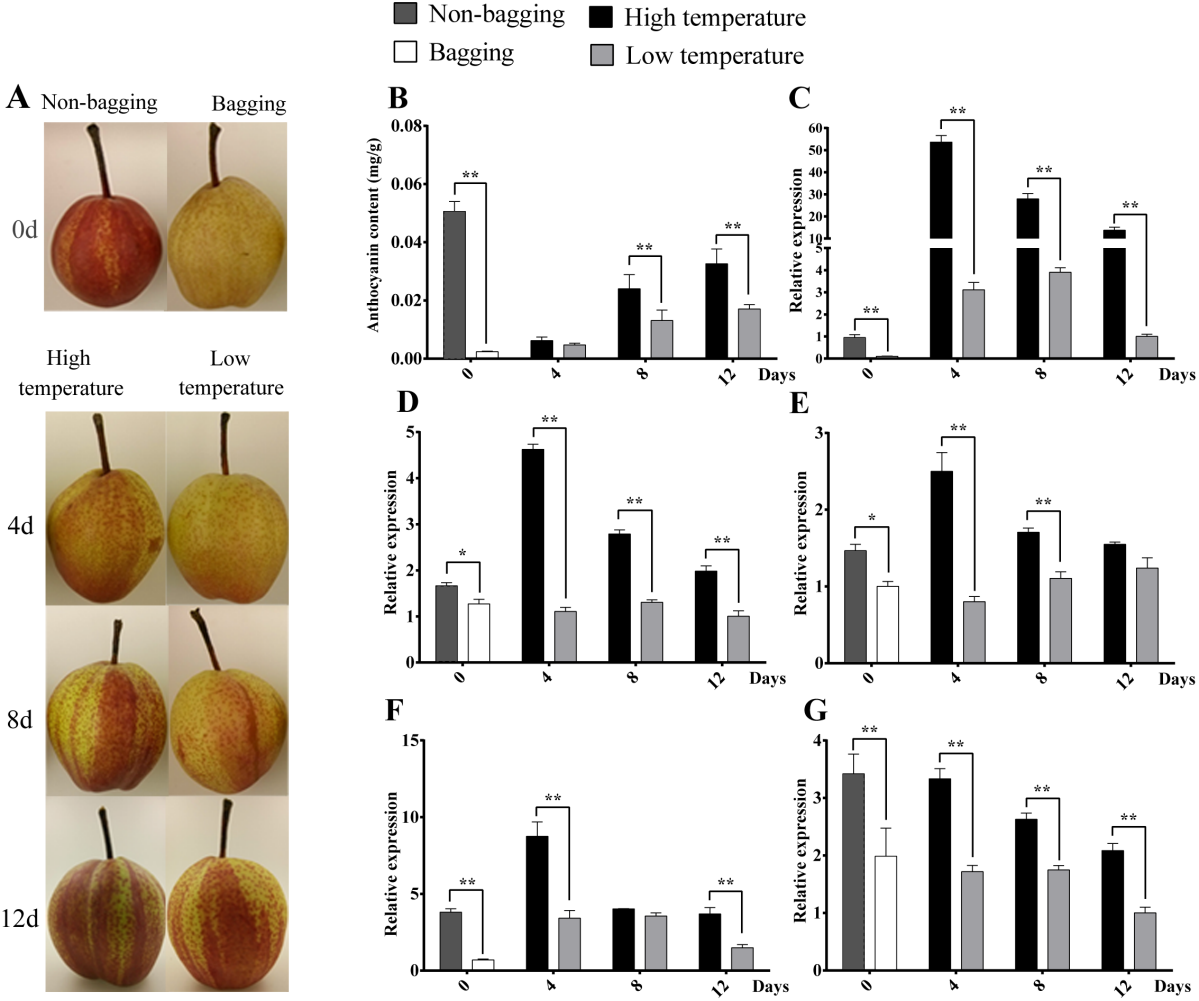
Skin color, anthocyanin content and expression analysis of related transcript factor genes in ‘Hongzaosu’ pears under different light and temperature conditions. ‘0’ indicated the bagged and non-bagged fruits when harvested at 15 days before commercial maturity. The fruit samples were collected at 4 d, 8 d and 12 d after debagging of bagging fruits with high temperature (HT) and low temperature (LT) treatment. Data marked with one and two stars indicated *P* < 0.05 and *P* < 0.01, respectively. (A) Skin color of pear fruit for different treatments. (B) Anthocyanin content of fruit for different treatments. (C), (D), (E), (F) and (G) indicated gene expression of *PbMYB10*, *PbbHLH3*, *PbWD40*, *PbrMADS11* and *PbrMADS12*, respectively.

In previous report, environmental factors, such as light and temperature, have been proposed to induce anthocyanin accumulation via the regulation of *bHLH*, *R2R3-MYB*, or small *R3-MYB* expression (Dubos et al., 2008; Cominelli et al., 2008; Olsen et al., 2009). To test the function of *PbrMADS11* and *PbrMADS12* involved in the regulation of anthocyanin synthesis, the anthocyanin content and expression pattern of *PbMYB10*, *PbbHLH3*, *PbWD40*, *PbrMADS11* and *PbrMADS12* genes in response to light and temperature were detected in the ‘Hongzaosu’ pear (Fig. 8). The red coloration of non-bagged fruit and yellow white color of bagged fruit were clearly seen when they were harvested at 15 days before commercial maturity. For anthocyanin content, non-bagged fruits were significantly higher than bagged fruits, it indicated that light promoted the fruit coloration. The expression levels of *PbMYB10*, *PbbHLH3*, *PbWD40*, *PbrMADS11* and *PbrMADS12* genes in non-bagged fruits were significantly higher than bagged fruits. It suggested that light promoted pear anthocyanin synthesis by up-regulating the expression of *PbrMADS11*, *PbrMADS12*, and other related genes.

**Figure 9:**
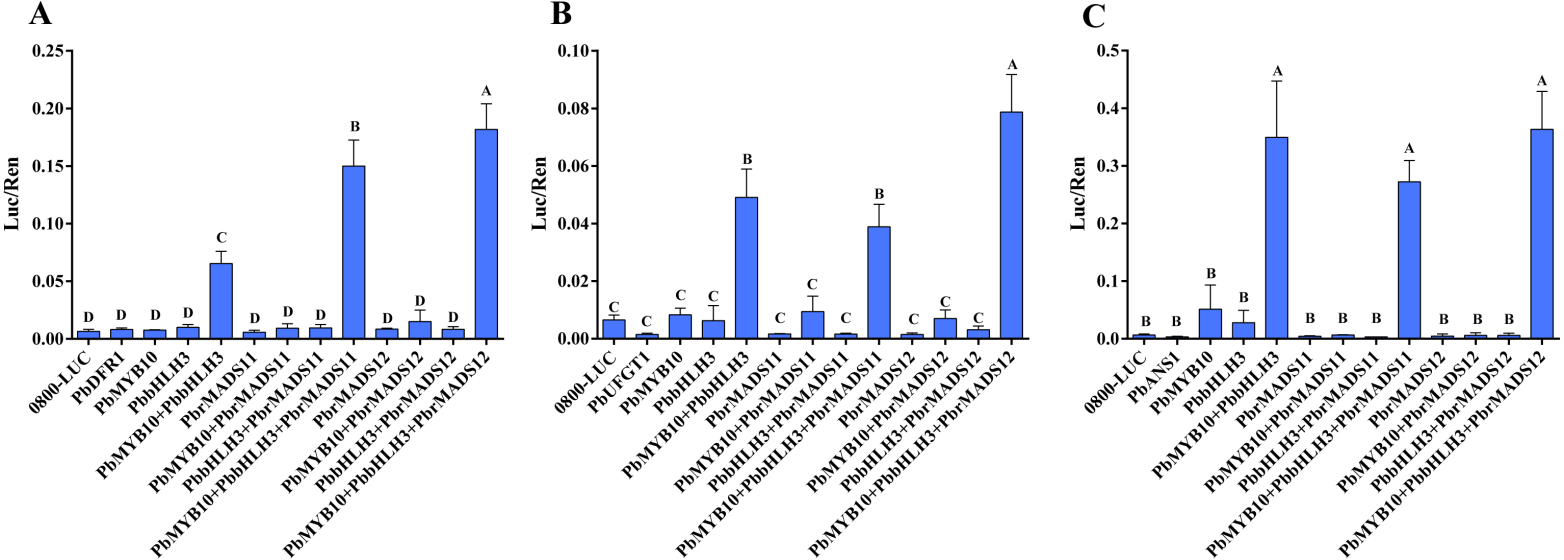
Effect of the *PbrMADS11* (*12*), *PbMYB10*, and *PbbHLH3* genes on activation of the promoter sequences of *PbDFR1*, *PbUFGT1*, and *PbANS1* genes. (A) *PbDFR1*, (B) *PbUFGT1*, (C) *PbANS1*. The dual luciferase assay shows promoter activity expressed as a ratio of promoter luciferase (LUC) to 35S Renilla (REN), where an increase in activity equates to an increase in LUC relative to REN. Each value represents the mean of three biological replicates. Different capital letters indicate significant differences among treatments (one-way ANOVA, least significant difference test at *P* < 0.01). Error bars show the standard deviation.

After de-bagging of bagged fruits, the coloration and anthocyanin content of fruits under high temperature (HT) and low temperature (LT) treatments all showed increasing trends (Fig. 9). However, it was interested to find that HT promoted higher anthocyanin content of pear than LT treatment, the significantly differences were detected at 8 d and 12 d treatment, which is different from previous report that LT is more effective than HT to promote the coloration and anthocyanin synthesis (Christie, Alfenito & Walbot, 1994; Ubi et al., 2006; Yamane et al., 2006; Zhang et al., 2012a). The similar phenomenon was also detected in ‘Yunhongli No. 1’ pear (Zhang et al., 2012b) and ‘Jonathan’ apple (Arakawa, 1991). The difference might due to genotype or species-specific response to environmental factors. The expression levels of *PbMYB10*, *PbbHLH3*, *PbWD40*, *PbrMADS11* and *PbrMADS12* genes under HT were significantly higher than LT in most developmental stages. The significant differences of gene expression were detected at 4d between HT and LT treatment, while the anthocyanin content showed significant difference at 8 d. It indicated the early starting of related gene expression and the lag behind of anthocyanin synthesis and accumulation. The similar phenomenon has also been observed in previous report (Kim et al., 2003; Zhang et al., 2012b). Based on all above analysis, it suggested that *PbrMADS11* and *PbrMADS12* involved in the regulation of anthocyanin synthesis response to light and temperature changes.

Through searching the promoter region (3,000 bp up-stream of ATG) of the seven structural genes by online software PLACE (Plant Cisacting Regulatory DNA Elements) (Higo et al., 1999), three types of MADS-binding cis-motifs (C(A/T)_8_G, CC(A/T)_8_GG, and CC(A/T)_6_GG) were detected in these genes (Fig. S8) (De Folter & Angenent, 2006). Thus, we speculate the possible mechanism of *MADS-box* transcription factors, that could bind to the promoter of structural genes and regulate their expression. In order to verify the predicted gene function of *MADS-box* transcription factors in the anthocyanin biosynthesis pathway of pear, further dual luciferase analyses were conducted. The *DFR* and *ANS* genes were indicated as limiting genes for anthocyanin biosynthesis in red-skinned ‘Zaobaimi’ pear in previous report (Zhang et al., 2011). The *ANS* and *UFGT* genes were also indicated as decisive genes for anthocyanin biosynthesis in six red-skinned pear cultivars with different genetic backgrounds in our previous report (Yang et al., 2014). The *DFR*, *ANS* and *UFGT* genes are key enzymes at later steps of anthocyanin biosynthesis pathway (Holton & Cornish, 1995; Fischer et al., 2007). Furthermore, *UFGT* and *DFR* promoters could be activated by *PbMYB10* gene, indicating their important roles in anthocyanin synthesis (Wang et al., 2013; Feng et al., 2015; Zhai et al., 2016). Therefore, the interaction of *PbrMADS11,12* genes with the promoter sequences of the *PbDFR1* (*Pbr005931.1*), *PbANS1* (*Pbr001543.2*), and *PbUFGT1* (*Pbr039986.1*) genes in transiently transfected *Arabidopsis* mesophyll protoplasts were evaluated (Fig. 9). The results showed that *PbrMADS11* and *PbrMADS12* could significantly improve promoter activity of *PbDFR1* when it was co-transformed with *PbbHLH3* and *PbMYB10*, with the promoting function of *PbrMADS12* higher than *PbrMADS11*. A similar promoting function was identified in *PbrMADS12* for *PbUFGT1*, while no promoting function was identified in *PbrMADS11*. However, neither *PbrMADS11* nor *PbrMADS12* showed a promoting function for the promoter of *PbANS1*, compared with co-transformed *PbbHLH3* and *PbMYB10.* Taken together, these results revealed that *PbrMADS12* gene together with *PbbHLH3* and *PbMYB10* partners, was able to activate the promoters of the *PbDFR1* and *PbUFGT1* genes in the anthocyanin pathway, while *PbrMADS11* could only activate *PbDFR1*.

## Conclusion

In this study, we provide genome-wide characterization and analysis of the *MADS-box* transcription factor family in pear. A total of 95 *MADS-box* genes were identified and classified into two types: type I and type II, according to the pre-classification trees. The pear type I *MADS-box* genes could be divided into three subfamilies, Mα, Mβ, and Mγ, and the type II *MADS-box* genes were further divided into 14 subfamilies. Within type II, 23 special proteins without K domain with conserved MADS domains and similarities to structural features of type I proteins were identified. Synteny analysis suggested that WGD or segmental duplication played critical roles in the expansion of *MADS* family in pear. Purifying selection was the major force driving the *PbrMADS* gene family, and one gene pair presented three positive codon sites. Further experimental analysis provided full-scale expression information for *PbrMADS* genes in vegetative and reproductive organs. Finally, *PbrMADS12* gene, together with *PbbHLH3* and *PbMYB10* partners, was confirmed to activate the promoters of the *PbDFR1* and *PbUFGT1* genes in the anthocyanin pathway, while *PbrMADS11* could only activate *PbDFR1*. The *PbrMADS11* and *PbrMADS12* were deduced involving in the regulation of anthocyanin synthesis response to light and temperature changes. In conclusion, the data and analysis generated in our study will facilitate further functional research, especially the study of pigmentation-related *MADS-box* genes.

##  Supplemental Information

10.7717/peerj.3776/supp-1 Supplemental Information 1 All supplementary tables Supplementary tables 1-7.

10.7717/peerj.3776/supp-2 Figure S1 Phylogenetic tree of type I *MADS-box* transcription factors in pear and *Arabidopsis* A total of 39 type I *MADS-box* proteins in pear, and 61 in *Arabidopsis* were used to construct the NJ tree. The subgroups are indicated by different branch colors.

10.7717/peerj.3776/supp-3 Figure S2 Maximum-likelihood trees of type I and II *MADS-box* transcription factors in pear (A) Tree of type II genes: a total of 56 type II *MADS-box* proteins in pear, 46 in *Arabidopsis*, and 8 in rice were used to construct the ML tree. The subgroups are indicated by different branch colors. (B) Tree of type I genes: a total of 39 type I *MADS-box* proteins in pear and 61 in *Arabidopsis* were used to construct the ML tree. The subgroups are indicated by different branch colors.

10.7717/peerj.3776/supp-4 Figure S3 Conserved motif compositions of pear *MADS-box* genes Different motifs are represented by different colored boxes. Motif 1 and motif 2 represent the *MADS* domain; Motifs 4, 7, and 9 are three fragments of the K domain. Box length represents motif length. Gene names, corresponding *P*-value and subgroup are shown on the left of figure. To better observe original motif distributions of different subfamilies, 23 non-K domain genes were removed from original subgroup and grouped. They are highlighted by a rectangle.

10.7717/peerj.3776/supp-5 Figure S4 Ka/Ks ratios of duplicated genes The boxplot shows the average value (black line in box), median value (red line in box), 1%, 25%, 75% and 99% value lines (box lines) of each data set. Double asterisks indicate significant differences between groups (*P* < 0.01).

10.7717/peerj.3776/supp-6 Figure S5 ML tree of pear and apple *MADS-box* genes for the branch site model Curves indicate segmental or tandem duplicated gene pairs. Node for each paralogous pair marked by star symbol was designated as the foreground branch and the others as background branches, respectively.

10.7717/peerj.3776/supp-7 Figure S6 Phylogenetic tree of anthocyanin biosynthesis-related genes and type II *PbrMADS* genes The tree was constructed using MEGA6.

10.7717/peerj.3776/supp-8 Figure S7 Anthocyanin content and coloration of fruit in the ‘Starkrimson’ and green strain at 40, 55, 70, and 85 DAFB

10.7717/peerj.3776/supp-9 Figure S8 *In silico* cis-element prediction of seven anthocyanin biosynthesis genes

## Additional Information and Declarations

### Data Availability

The following information was supplied regarding data availability: The raw data has been supplied as Supplemental Files.
