# Supplementary material for: Genome-wide identification of the MADS-box transcription factor family in pear (Pyrus bretschneideri) reveals evolution and functional divergence
Source: PeerJ. 2017 Sep 11;5:e3776. doi: 10.7717/peerj.3776 (PMC5598432; doi:10.7717/peerj.3776)
Supplement: Figure S2 — (A) Tree of type II genes: a total of 56 type II MADS-box proteins in pear, 46 in Arabidopsis, and 8 in rice were used to construct the ML tree. The subgroups are indicated by different branch colors. (B) Tree of type I genes: a total of 39 type I MADS-box proteins in pear and 61 in Arabidopsis were used to construct the ML tree. The subgroups are indicated by different branch colors. [file peerj-05-3776-s003.pdf]

(a)

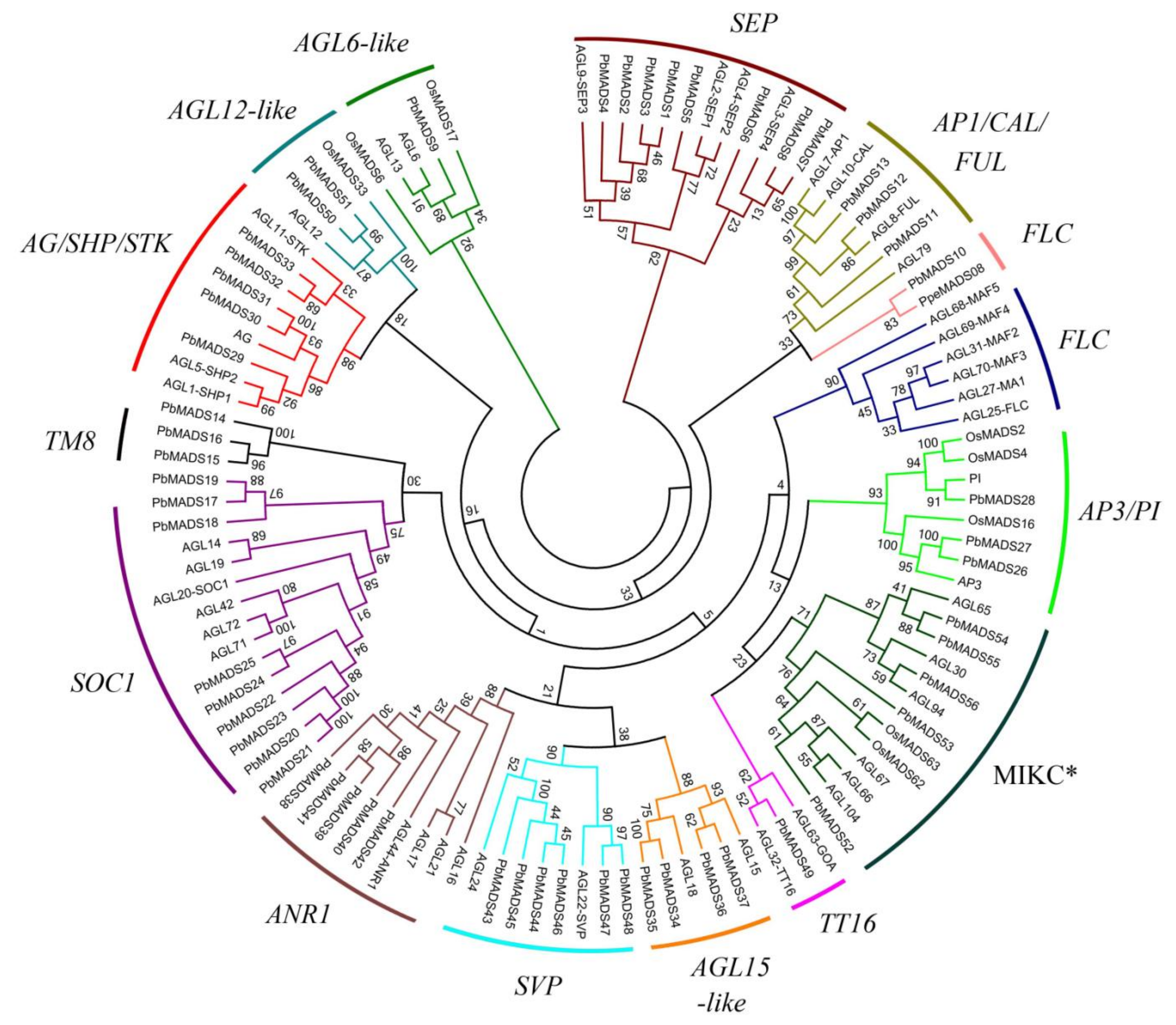

(b)

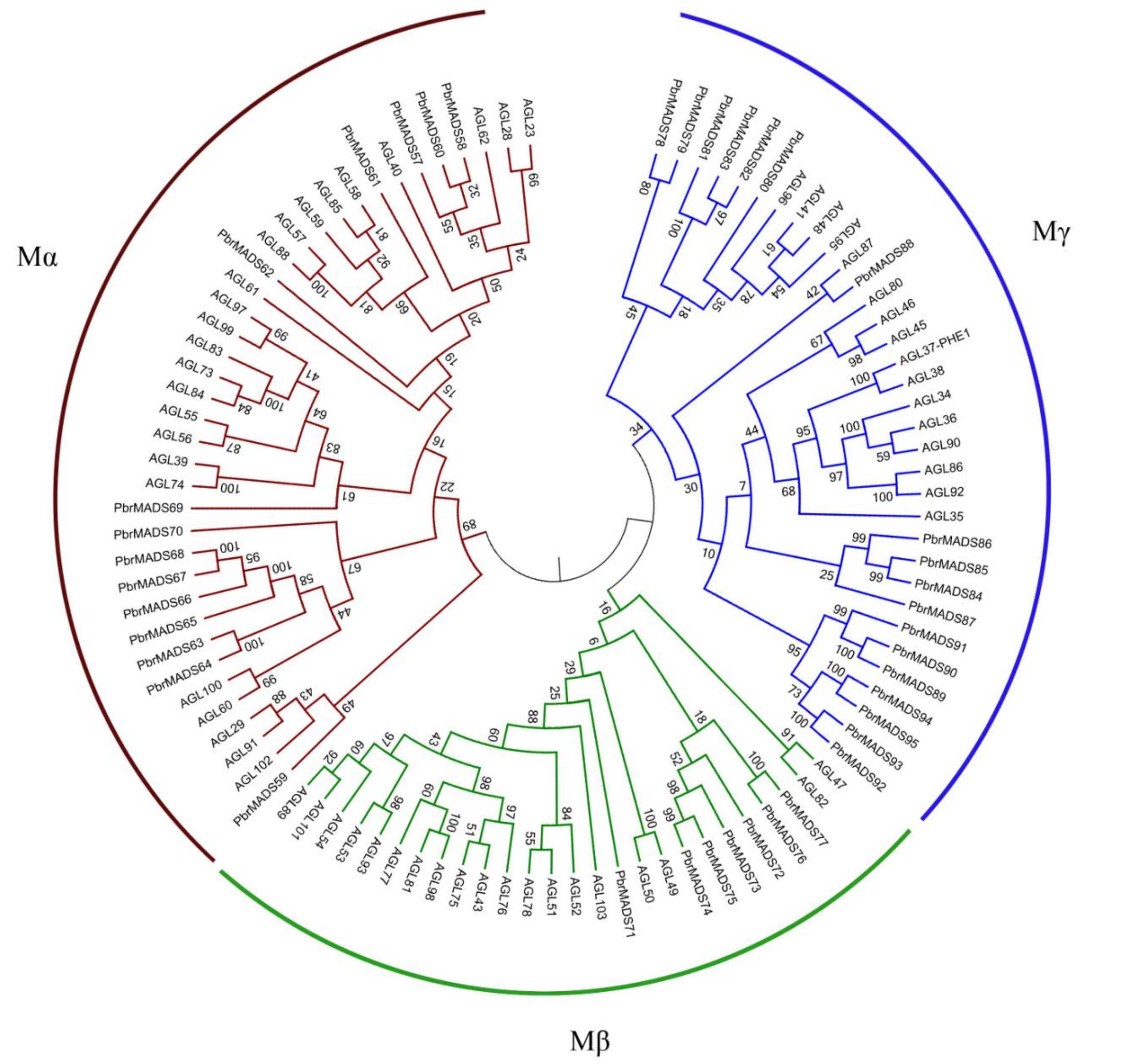

Supplementary figure 2. Maximum likelihood trees of type I and II *MADS-box* transcription factors in pear. (a) Tree of type II genes: a total of 56 type II *MADS-box* proteins in pear, 46 in *Arabidopsis*, and 8 in rice were used to construct the ML tree. The subgroups are indicated by different branch colors. **(b)** Tree of type I genes: a total of 39 type I *MADS-box* proteins in pear and 61 in *Arabidopsis* were used to construct the ML tree. The subgroups are indicated by different branch colors.
