# Supplementary material for: Genome-wide identification of the MADS-box transcription factor family in pear (Pyrus bretschneideri) reveals evolution and functional divergence
Source: PeerJ. 2017 Sep 11;5:e3776. doi: 10.7717/peerj.3776 (PMC5598432; doi:10.7717/peerj.3776)
Supplement: Figure S3 — Different motifs are represented by different colored boxes. Motif 1 and motif 2 represent the MADS domain; Motifs 4, 7, and 9 are three fragments of the K domain. Box length represents motif length. Gene names, corresponding P-value and subgroup are shown on the left of figure. To better observe original motif distributions of different subfamilies, 23 non-K domain genes were removed from original subgroup and grouped. They are highlighted by a rectangle. [file peerj-05-3776-s004.pdf]

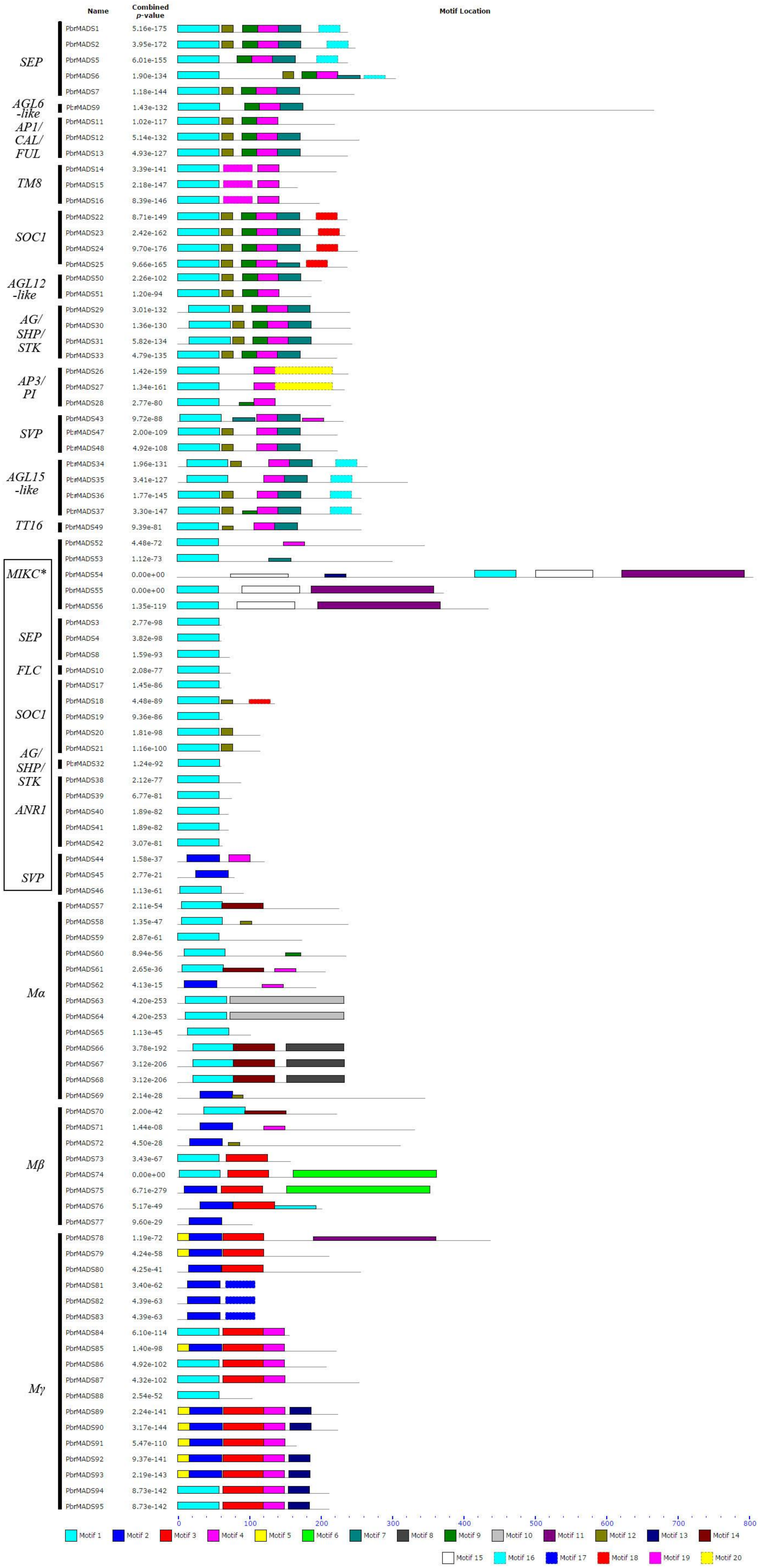

Supplementary figure 3. Conserved motif compositions of pear *MADS-box* genes. Different motifs are represented by different colored boxes. Motif 1 and motif 2 represent the MADS domain, Motifs 4, 7, and 9 are three fragments of the K domain. Box length represents motif length. Gene names, corresponding *P*-value and subgroup are shown on the left of figure. To better observe original motif distributions of different subfamilies, 23 intermediate form genes were removed from original subgroup and grouped. They are highlighted by a rectangle.
