# Supplementary material for: Genome-wide identification of the MADS-box transcription factor family in pear (Pyrus bretschneideri) reveals evolution and functional divergence
Source: PeerJ. 2017 Sep 11;5:e3776. doi: 10.7717/peerj.3776 (PMC5598432; doi:10.7717/peerj.3776)
Supplement: Figure S4 — The boxplot shows the average value (black line in box), median value (red line in box), 1%, 25%, 75% and 99% value lines (box lines) of each data set. Double asterisks indicate significant differences between groups (P < 0.01). [file peerj-05-3776-s005.pdf]

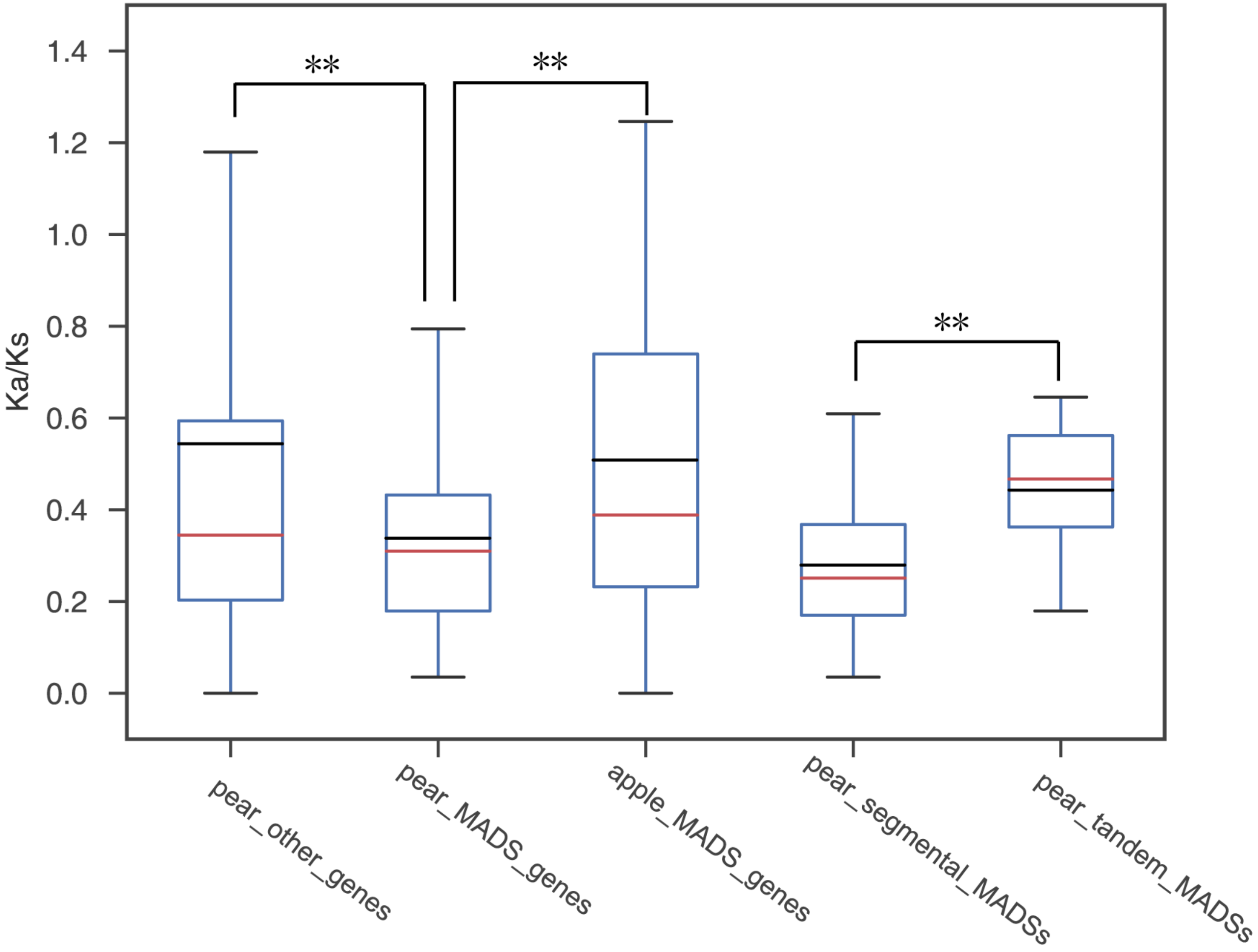

Supplementary figure 4. Ka/Ks ratios of duplicated genes. The boxplot shows the average value (black line in box), median value (red line in box), 1%, 25%, 75% and 99% value lines (box lines) of each data set. Double asterisks indicate significant differences between groups ($P < 0.01$).
