# Supplementary material for: Genome-wide identification of the MADS-box transcription factor family in pear (Pyrus bretschneideri) reveals evolution and functional divergence
Source: PeerJ. 2017 Sep 11;5:e3776. doi: 10.7717/peerj.3776 (PMC5598432; doi:10.7717/peerj.3776)
Supplement: Figure S5 — Curves indicate segmental or tandem duplicated gene pairs. Node for each paralogous pair marked by star symbol was designated as the foreground branch and the others as background branches, respectively. [file peerj-05-3776-s006.pdf]

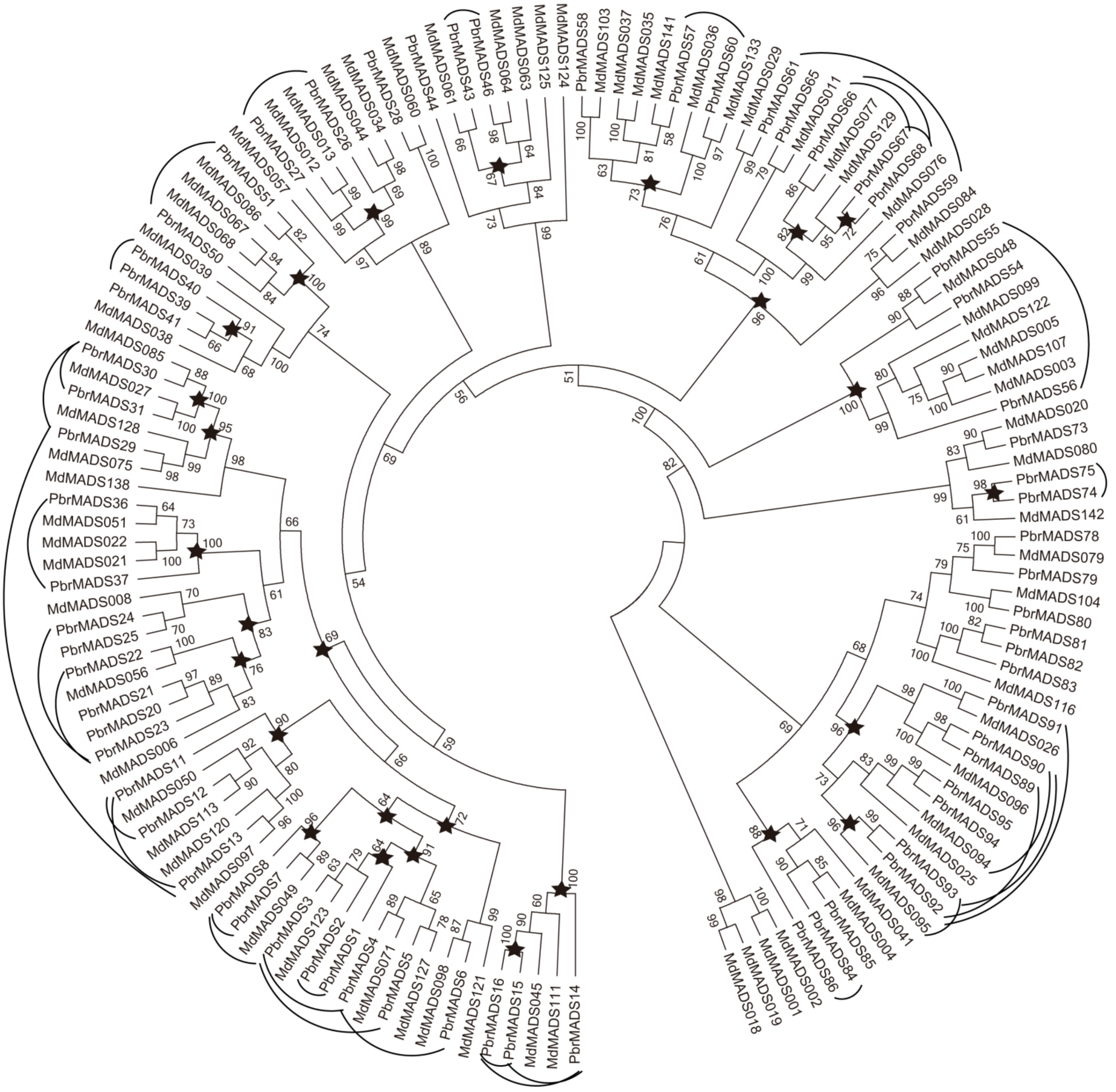

Supplementary figure 5. ML tree of pear and apple *MADS-box* genes for the branch site model. Curves indicate segmental or tandem duplicated gene pairs. Node for each paralogous pair marked by star symbol was designated as the foreground branch and the others as background branches, respectively.
