# Supplementary material for: Genome-wide identification of the MADS-box transcription factor family in pear (Pyrus bretschneideri) reveals evolution and functional divergence
Source: PeerJ. 2017 Sep 11;5:e3776. doi: 10.7717/peerj.3776 (PMC5598432; doi:10.7717/peerj.3776)
Supplement: Figure S6 — The tree was constructed using MEGA6. [file peerj-05-3776-s007.pdf]

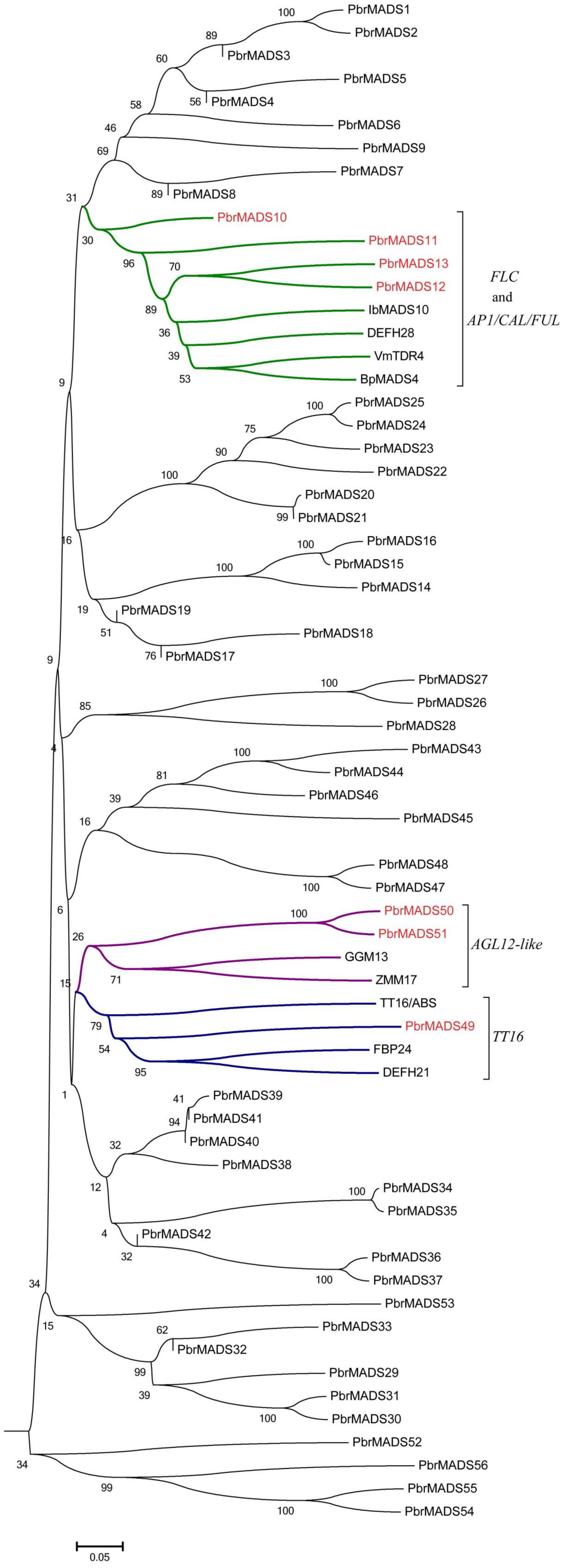

Supplementary figure 6. Phylogenetic tree of anthocyanin biosynthesis-related genes and type II *PbrMADS* genes. The tree was constructed using MEGA6.
