# Supplementary material for: Genome-wide identification of the MADS-box transcription factor family in pear (Pyrus bretschneideri) reveals evolution and functional divergence
Source: PeerJ. 2017 Sep 11;5:e3776. doi: 10.7717/peerj.3776 (PMC5598432; doi:10.7717/peerj.3776)
Supplement: Figure S8 [file peerj-05-3776-s009.pdf]

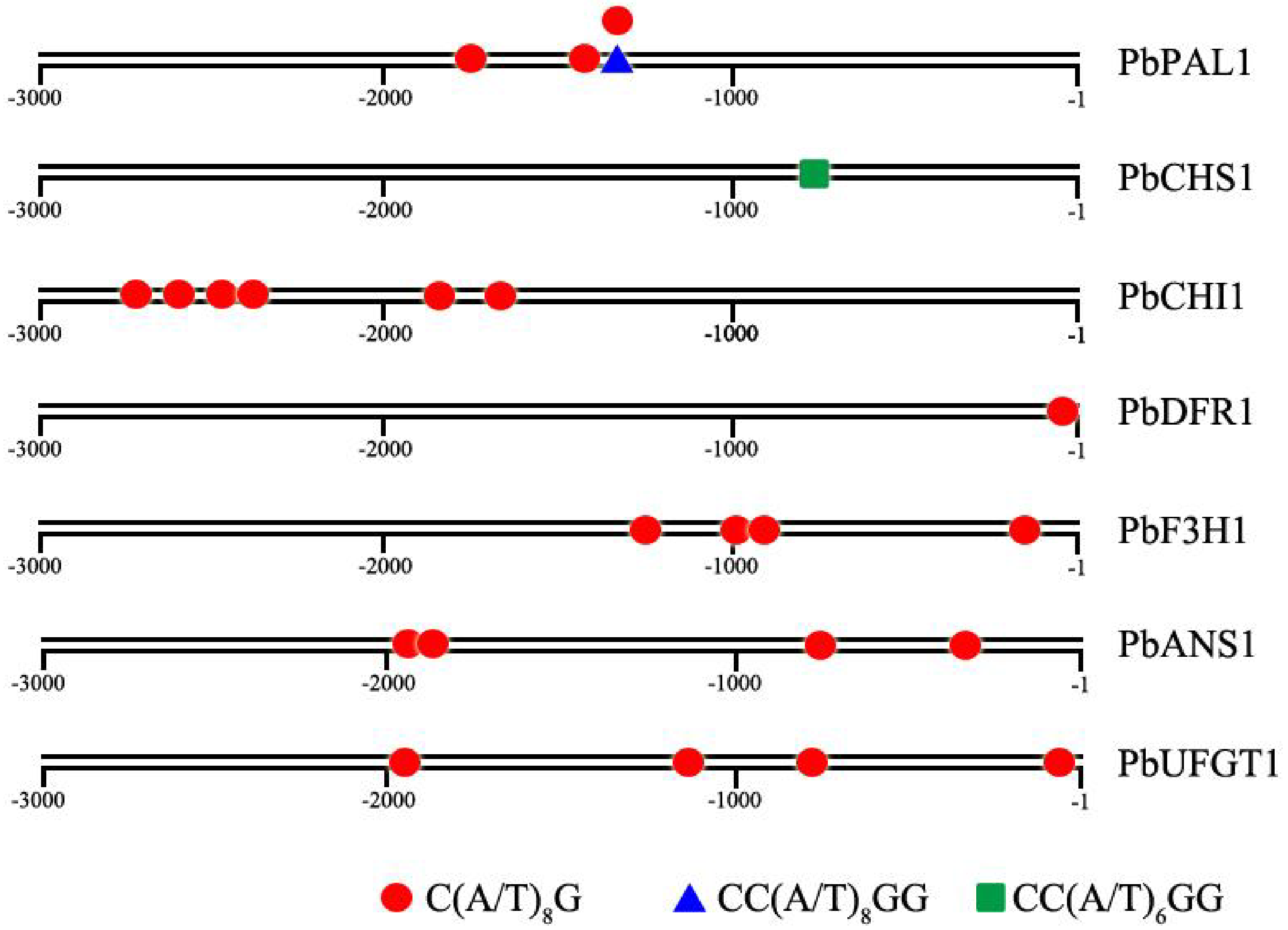

Supplementary figure 8. ***In silico*** **cis-element prediction of seven anthocyanin biosynthesis genes.**
