# Supplementary material for: Genome-wide identification of the MADS-box transcription factor family in pear (Pyrus bretschneideri) reveals evolution and functional divergence
Source: PeerJ. 2017 Sep 11;5:e3776. doi: 10.7717/peerj.3776 (PMC5598432; doi:10.7717/peerj.3776)
Supplement: Figure S1 — A total of 39 type I MADS-box proteins in pear, and 61 in Arabidopsis were used to construct the NJ tree. The subgroups are indicated by different branch colors. [file peerj-05-3776-s002.pdf]

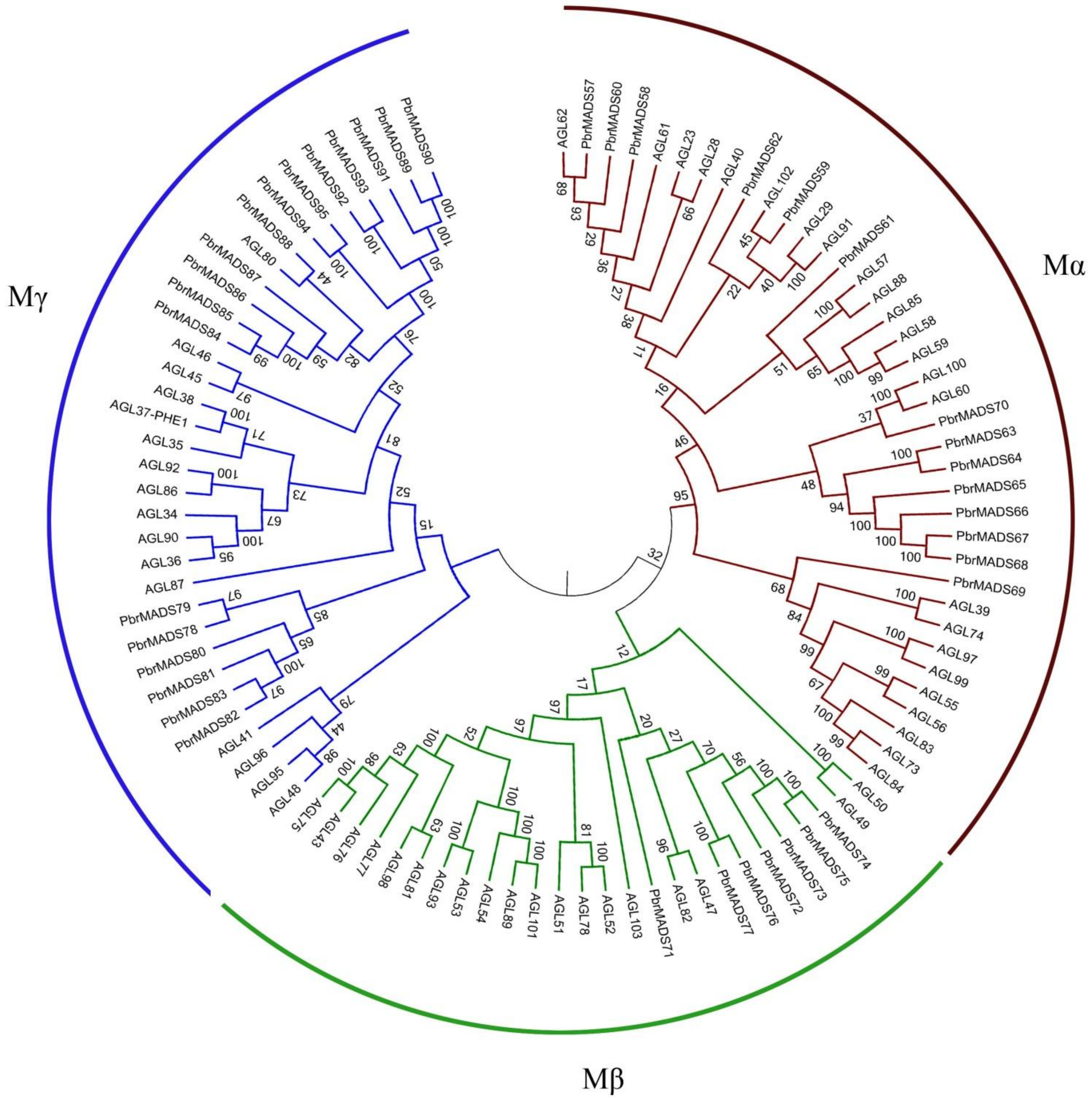

Supplementary figure 1. Phylogenetic tree of type I *MADS-box* transcription factors in pear and *Arabidopsis*. A total of 39 type I *MADS-box* proteins in pear and 61 in *Arabidopsis* were used to construct the NJ tree. The subgroups are indicated by different branch colors.
